# How effective are Fatigue Risk Management Systems (FRMS)? A review

**DOI:** 10.1016/j.aap.2021.106398

**Published:** 2021-10-28

**Authors:** Madeline Sprajcer, Matthew J.W. Thomas, Charli Sargent, Meagan E. Crowther, Diane B. Boivin, Imelda S. Wong, Alison Smiley, Drew Dawson

**Affiliations:** a Appleton Institute, CQUniversity, Adelaide, Australia; b Douglas Mental Health University Institute, McGill University, Montreal, Canada; c Centers for Disease Control and Prevention/National Institute for Occupational Safety and Health, USA; d Human Factors North Inc, Canada

**Keywords:** Sleep, Risk, Safety, Fatigue risk management systems, FRMS, Fatigue

## Abstract

**Objective:**

Fatigue Risk Management Systems (FRMS) are a data-driven set of management practices for identifying and managing fatigue-related safety risks. This approach also considers sleep and work time, and is based on ongoing risk assessment and monitoring. This narrative review addresses the effectiveness of FRMS, as well as barriers and enablers in the implementation of FRMS. Furthermore, this review draws on the literature to provide evidence-based policy guidance regarding FRMS implementation.

**Methods:**

Seven databases were drawn on to identify relevant peer-reviewed literature. Relevant grey literature was also reviewed based on the authors’ experience in the area. In total, 2129 records were screened based on the search strategy, with 231 included in the final review.

**Results:**

Few studies provide an evidence-base for the effectiveness of FRMS as a whole. However, FRMS components (e.g., bio-mathematical models, self-report measures, performance monitoring) have improved key safety and fatigue metrics. This suggests FRMS as a whole are likely to have positive safety outcomes. Key enablers of successful implementation of FRMS include organisational and worker commitment, workplace culture, and training.

**Conclusions:**

While FRMS are likely to be effective, in organisations where safety cultures are insufficiently mature and resources are less available, these systems may be challenging to implement successfully. We propose regulatory bodies consider a hybrid model of FRMS, where organisations could choose to align with tight hours of work (compliance) controls. Alternatively, where organisational flexibility is desired, a risk-based approach to fatigue management could be implemented.

## 1. Introduction

Fatigue Risk Management Systems (FRMS) are based on the systematic identification of workplace hazards relating to fatigue. These systems differ significantly from the often ad hoc approach to fatigue management found in traditional compliance-based systems, based solely on work hours. A fundamental component of FRMS is the identification of risk, rather than compliance. This is a key distinction, as compliance does not necessarily indicate fatigue, risk, or safety. Put simply, even if a roster complies with hours of work regulations, a worker may still experience fatigue. In contrast, FRMS allow appropriate risk-based control measures to be established and facilitate dynamic changes to work systems to promote safety. These proportionate control measures are determined based on an analysis of the likelihood of fatigue (based on observed fatigue, prior sleep, and sleep opportunity) combined with the consequences of a fatigue-related error or event (i.e., risk assessment) (Dawson and McCulloch, 2005).

Advisory and decision-making tools to support the hazard identification process are also typically included within FRMS (Butlewski et al., 2015). These tools can be integrated within existing safety management systems, where fatigue can be managed in a similar way to other operational risks (Stewart et al., 2006). Tools may include roster assessment software, systems for assessing individual levels of fatigue likelihood, fatigue reduction tools, and fatigue proofing strategies (Dawson and McCulloch, 2005; Lillington and McLeod, 2012).

There is some confusion over what is meant by the term FRMS. At one extreme it is a term appropriated by traditional prescriptive systems that merely limit the working time arrangement and add certain compliance requirements (e.g., training/education). Within these systems, risk is mitigated only by limiting hours and is not quantified via formal risk assessment approaches (e.g., ISO 31000). At the other extreme, fatigue is treated as one of many hazards to be managed systematically using standardised quantitative models of risk and safety management. Here, the working time arrangement is used to assess the likelihood of fatigue, the work task(s) is used to determine the consequence of a fatigue-related error. Based on this approach, the working time arrangement/task dyad are used principally to quantify the risk and changes to the working time arrangement to either increase or mitigate the risk. Under this approach, proportional control measures can be implemented without necessarily altering hours of work (Dawson and Bowe, 2019; Gander, 2015).

Despite a significant body of literature informing the development of FRMS, there has been no comprehensive review of the effectiveness of FRMS to date. As such, this narrative review aims to answer the following research questions:

1. How effective are FRMS in reducing fatigue and improving safety outcomes?
2. What are the key barriers and enablers to effective implementation of FRMS?
3. How should FRMS be implemented from a policy perspective?

## 2. Methods

### 2.1. Search strategy

The present review was designed around a systematic search strategy informing a subsequent narrative synthesis of the available peer-reviewed and grey literature. The process adopted in the current review adheres to the methodology as set out in the Cochrane Handbook for Systematic Reviews of Interventions (Higgins et al., 2021). Traditional peer reviewed literature was sourced from academic databases, including:

MEDLINE (Ovid)
PsycINFO
IEEE Xplore Digital Library
Scopus
Web of Science
MESH Occupational Health
NIOSHTIC II

An initial search strategy was determined based on Population, Intervention, Comparison, and Outcome (PICO) terminology. However, this strategy was over inclusive and had poor specificity (see Supplementary Material).

The below sets out the basic framework for the revised search strategy. From the initial strategy, the number of search terms was considerably reduced, but in practice, this approach provided a more effective search algorithm overall. Information on quality assessment can be seen below. The search strategy was condensed to use a modified PICO structure:

Population: Any organization that has described a fatigue-risk management intervention either discretely or as part of a more integrated FRMS.
Intervention: One or more fatigue risk management interventions including: 1) hazard identification; 2) risk assessment; or 3) control measures.
Comparison: No requirement for comparison data was included in the review but was noted where relevant.
Outcome: No requirement for outcome data to be included.

Search terms can be seen in Table 1. As can be seen in this table, fatigue management systems (e.g., fatigue management plans, programs, etc.) that are not clearly described as FRMS or include some components of FRMS have been included within the search. This is to ensure that all possible evaluative documents are included within the review.

The below provides an example of the Boolean construction of the search algorithm as required for searching using the Scopus database.

(TITLE-ABS-KEY (“Work Health and Safety” OR “Health” OR “Well-being” OR “Safety” OR “Injury” OR “Accident” OR “Risk”)) AND (TITLE-ABS-KEY (“Fatigue management” OR “Fatigue risk management system” OR “Fatigue management plan” OR “Fatigue management program” OR “Fatigue risk” OR “Fatigue hazard”))

### 2.2. Grey literature search strategy

Grey literature, which is not typically found within standard databases, was not accessible using the search strategy described above. As such, the authors were unable to perform a systematic search. However, the authors’ experience in the area and work with regulators and operators world-wide (including in areas such as rail, healthcare, and mining) facilitated access to a number of documents describing various elements of FRMS in practice.

Due to the nature of the strategy to identify relevant grey literature, there may be a risk of bias – that is, there may be other examples of grey literature that the authors were unaware of and thus did not include in this review. Grey literature provided significant evidence otherwise not available for this review and therefore, the search for and inclusion of grey literature was deemed necessary. The risk of bias associated with grey literature is an unavoidable limitation (in that there are no relevant databases that could be searched systematically) in the present in review. Further bias and quality assessment information can be seen in the Supplementary Materials.

### 2.3. Eligibility

**Fig. 1.**
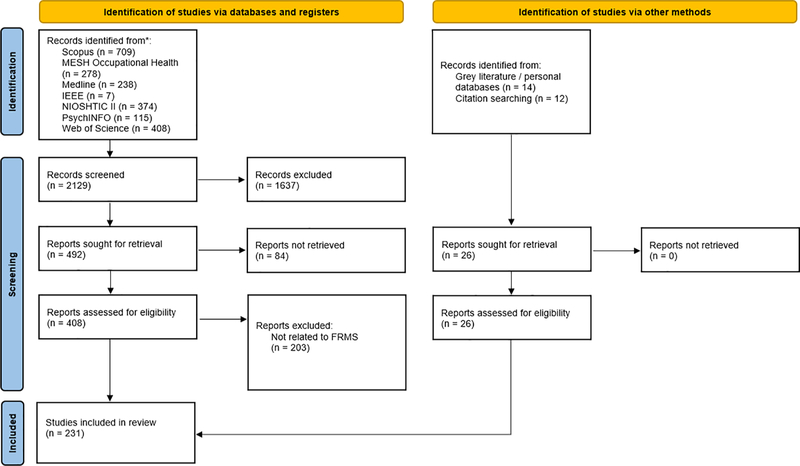
Search and screening process.

Any documents that focused explicitly on FRMS were included for analysis, even if descriptive in nature. Addressing FRMS was used as the primary criteria used to assess the initial 2,129 documents (see Fig. 1). Documents excluded at this point did not relate to fatigue management in any way. Documents were included if they addressed interventions that form the foundations of FRMS. Interventions were defined in terms of: 1) a specific hazard identification process (e.g., bio-mathematical modelling of rosters); 2) a specific risk assessment process (e.g., task analysis or incident investigation); or 3) a specific control of fatigue-related risk (e.g., napping policy/facilities). Therefore, the primary inclusion criterion was being peer-reviewed or grey literature that described one or more FRMS components. As the research questions included barriers to, and enablers of, implementation of FRMS and policy implications, documents were included in the review even if safety or productivity outcomes were not presented. Additional inclusion and exclusion criteria included:

Date: 1990 – Present
Language: English and French
Study type: No exclusions (as above – descriptive papers/reports were not excluded)
Type of participants: Any worker/workplace
Type of intervention: Any fatigue management intervention
Outcome measures: Any relating to safety, productivity, worker health and well-being

The screening process was undertaken by two authors (MS and MC) and reviewed by a third author (MT). Pilot screening was done via consensus. Screening in this manner was done at both the title and abstract stage and the full text stage. Documents included and excluded in each stage of screening can be seen in Fig. 1. This review was not registered.

### 2.4. Included documents

Seven databases were used to identify literature, in addition to the authors’ personal collections and industry contacts. A total of 2129 records were initially produced from the searches conducted with each database (Scopus: 709; MESH Occupational Health: 278; Medline: 238; IEEE: 7; NIOSHTIC II: 374; PsychINFO: 115; Web of Science: 408). Titles and abstracts were screened for eligibility according to the inclusion/exclusion criteria and a total of 204 papers were identified for full review. In addition, 26 examples of grey literature and academic publications were included based on the authors’ knowledge and collections of literature.

Due to the significant number of publications included in this review, a full reference list is provided in the Supplementary Materials, along with additional information regarding the search strategy.

## 3. Results

### 3.1. Evaluation of fatigue management interventions

Of the relevant documents, 92 (40%) provided purely descriptive information and/or regulatory advice/guidelines, while the remaining documents present information addressing certain components of FRMS. Of the documents that present an evaluation of an entire fatigue management system, several (n = 5) provided an evaluation of FRMS or fatigue management programs (FMP) as a whole (Barger et al., 2017; Dara, 2019; Fourie et al., 2010; Lamp et al., 2019; Smiley et al., 2010). Based on the issues identified above regarding the classification of systems as FRMS (i.e., where some systems are self-described as FRMS, but do not include fatigue risk assessment processes), we have included both FRMS (which include a fatigue risk assessment component) and fatigue management plans (which, while including certain aspects of FRMS, do not include a risk assessment component). As such, not all of these five documents address all FRMS components – but instead provide evaluation of fatigue management programs and/or systems. In particular, a risk assessment framework (arguably the core component of FRMS) was either was not evaluated (Smiley et al., 2010), or was not *explicitly* included in the document (Barger et al., 2017; Dara, 2019). Just two documents included this critical risk assessment component of FRMS (Fourie et al., 2010; Lamp et al., 2019). Relevant data from each of these studies has been extracted and is presented in Table 2.

Of the five documents that provided FRMS or FMP evaluation, three report data pre- and post- FRMS/FMP implementation. Data are presented in different employee populations, including firefighters (Barger et al., 2017), nurses (Dara, 2019), and professional drivers (Smiley et al., 2010). The study performed with Australian firefighters included the random allocation of fire stations to either a fatigue risk management program or no additional program (Barger et al., 2017). Findings from this survey data suggest that sleep was significantly improved in the intervention group, as was daytime sleepiness. Participants also reported finding the program “important and helpful” with improvements to their personal sleep hygiene. However, these findings are based on self-report measures only, and do not include standardized, objective, safety metrics (e.g., incidents, errors, etc.). As such, while we can cautiously interpret these findings as supportive of the use of FRMS, objective outcomes and operational/safety impacts must be included in evaluations. Objective measures of sleep and wake behaviour (in this case actigraphy) were collected in a study of surgical intensive care nurses (Dara, 2019). However, this document was a conference abstract, and did not report on these objective sleep measures. This study did demonstrate significant improvement in self-reported fatigue and sleepiness after FRMS implementation among nurses, which aligns with the findings observed with firefighters (Barger et al., 2017). Taken together, these findings suggest that at a minimum, FRMS is likely to improve self-reported fatigue outcomes in workers.

The only study to objectively evaluate an FMP (though not an FRMS) within a pre- and post- implementation framework was done in professional drivers in Canada and the United States (Smiley et al., 2010). Pre- and post- implementation data were collected via personal digital assistant devices and sleep/wake information as obtained via actigraphy and at home sleep screening. Safety, health, and operational data were also provided by the relevant participating organisations. As with the previously discussed studies, sleep improved significantly post-implementation, as determined by both self-report and actigraphically measured sleep. Furthermore, fewer safety events (e.g., microsleeps, near misses, road infractions, and accidents) were recorded post-implementation and participants reported improved fatigue management activities (e.g., education, alertness management strategies) as part of FRMS implementation. As with the previously described studies that evaluated FRMS as a whole, this study strongly suggests that FRMS is effective in improving sleep, fatigue, and safety outcomes – though it is clear that further research is warranted.

Though not employing a pre- and post-implementation design, one study compared aviation safety outcomes between two groups of flights – one group where FRMS had been implemented, and one where fatigue was managed via safety standard operation (i.e., prescriptive fatigue management as opposed to risk-based fatigue management) (Lamp et al., 2019). This study found no differences in safety outcomes between the two systems of work. Outcome measures of this study included in flight sleep obtained by crew, cognitive performance measures, and self-reported fatigue/sleepiness. This study suggests that at a minimum, FRMS is as safe as existing prescriptive fatigue management systems. However, as aviation can be considered an ‘ultra-safe’ industry (Stoop, 2017), these similarities may reflect an overall low level of safety–critical events.

An additional study that provided an evaluation of FRMS as a whole used a qualitative methodology (interviews) to obtain worker perspectives on FRMS post-implementation (Fourie et al., 2010). These interviews indicated that FRMS resulted in positive outcomes such as improved self-reported safety, morale, and competitive advantage. However, the number of organisations who reported each of these outcomes was moderate (2–6 organisations reporting each positive finding) in comparison to the total organisation pool (n = 12). This suggests that FRMS implementation may have mixed results, potentially depending on industry and/or organisational differences (e.g., size, complexity, cultural maturity). However, as with the findings of the other four documents that outline FRMS evaluation, further research is necessary – both to understand why these differences in efficacy are seen, and to see outcomes based on objective measures (e.g., safety metrics, objectively measured and reported sleep outcomes, etc.).

In total, 117 documents (53%) present peer reviewed studies involving certain aspects of FRMS or FMP (in addition to five studies that evaluated FRMS or FMP as a whole), while 70 documents included academically-based reviews and/or peer reviewed guidance in the area of fatigue management published in standard academic journals. Industry guidelines (n = 17) and regulations/legislation (n = 2) made up an additional 8% of included documents. Three published case studies (1%), and 17 industry conference presentations (7%) were included. The majority of these industry conference presentations were presented by management level employees of organisations where FRMS was either considered or implemented. Documents within this review cover FRMS and associated components in a variety of industries, primarily including healthcare, aviation, and transport. Additional industries mentioned less frequently include emergency services (e.g., firefighters, police), oil and gas, mining, and maritime operations. Many documents did not focus on one specific industry, however, instead providing reviews or studies investigating FRMS (or a component thereof) more broadly.

An evaluation strategy was designed to assess the documents identified in this review due to the paucity of documents which evaluated FRMS as a whole. This strategy involved assigning each document to a category representing the type of evaluation or evidence provided. Categories are presented in Table 3.

**Fig. 2.**
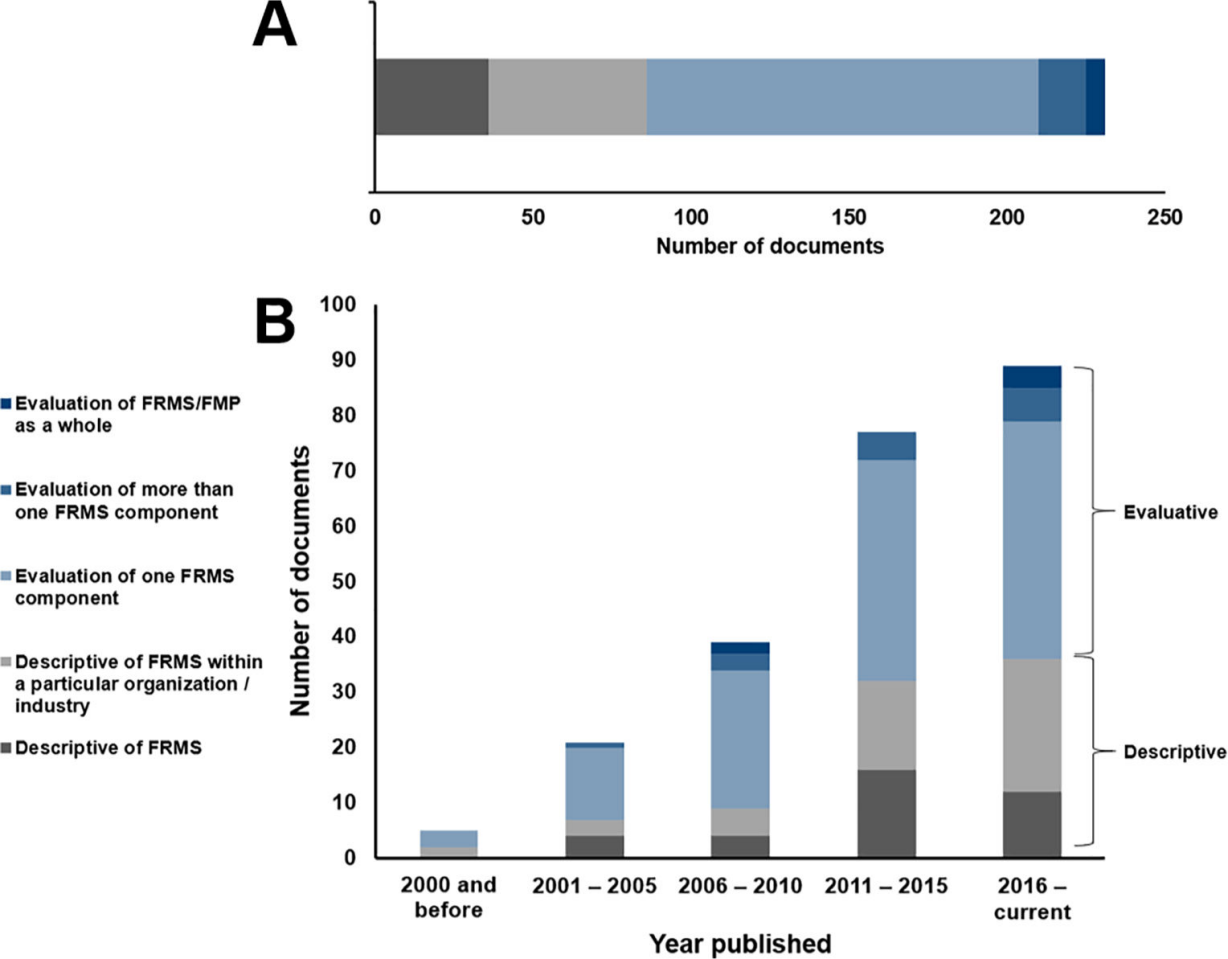
Published documents providing description and evaluation of FRMS. Panel A presents the total number of documents published in each category. Panel B presents the included documents over time.

Each document was assigned a category as illustrated in Fig. 2. The figure also includes the year of publication (2000 and before, 2001–2005, 2006–2010, 2011–2015, 2016 – current). Over time, the number of documents published regarding FRMS has increased, but most documents are either solely descriptive or provide evaluation of just one FRMS component. Individual categories assigned to documents is provided in the Supplementary Materials.

### 3.2. Key FRMS components

The majority of documents included in this review provided descriptive information about FRMS and/or specific components of FRMS. Those that included an evaluation of a whole system evaluated both FRMS and FMP. FRMS components can be categorized as *predictive* (monitoring work schedules), *proactive* (monitoring real time worker fatigue and fitness for duty), or *reactive* (identifying the contribution of fatigue to safety events). The following key information was provided by the included body of work:

#### 3.2.1. Predictive FRMS controls

Predictive FRMS controls described by the documents included in the present review include hours of work rules, roster design and review, bio-mathematical modelling, and communication policies. These documents indicate that the use of predictive roster assessment is likely to result in lower levels of fatigue and fewer injuries/accidents (Anderson et al., 2013; Barger et al., 2007; Barger et al., 2009; Flynn-Evans et al., 2018; Gander et al., 2019; Goldenhar et al., 2003). This is because these roster assessments aim to avoid the increased likelihood of fatigue associated with extended shifts, shifts that occur during circadian low points (typically 0200–0500 h), and other non-standard work arrangements (Anderson et al., 2013; Barger et al., 2007; Cabon et al., 2012; Flynn-Evans et al., 2018; Gander et al., 2019; Nix and Brunette, 2015; Wong et al., 2018). Unsurprisingly, findings indicate that a consolidated night-time sleep opportunity is best for gaining sufficient sleep and maintaining subsequent effective work performance (Jackson et al., 2014). Furthermore, using roster assessment and modelling, populations of sailors and train drivers obtain adequate sleep within a fatigue-risk management program (Darwent et al., 2015; Thomas et al., 2019). Bio-mathematical models of fatigue were also covered by several key documents, and were found to be effective in predicting fatigue and managing fatigue-related risk (Fletcher and Dawson, 2001; Ingre et al., 2014; Li, 2016; Moore-Ede et al., 2004; Van Dongen et al., 2007). These models of fatigue provide important information for organisations and personnel, and allow for safer, evidence-based decision making (Dawson et al., 2011). Further, there is evidence models can be used as an alert system for employees who are likely to be experiencing fatigue (Reifman et al., 2016).

#### 3.2.2. Proactive FRMS controls

Proactive risk controls used within FRMS described within the documents included in the present review include individual fatigue assessment based on self-report, prior sleep wake behaviour, independent observation, and behavioural monitoring. Evidence suggests that the development of validated self-assessment tools within an FRMS allows for non-invasive, quick and affordable means of measuring or reporting fatigue (Arnaldo et al., 2016). This is of particular importance as it has been indicated that despite training, many employees lack the ability to self-report fatigue without the assistance of formal assessment tools (O’Connor and Lincoln, 2013). Performance indicators were also used to identify fatigue within an FRMS (van den Berg et al., 2015). Examples of effective performance indicators include hard-braking in drivers (Mollicone et al., 2019), drive/rest time diaries supported by odometer readings (Mirowski and Scanlan, 2018), and measures such as psychomotor vigilance task performance (Wu, 2013; Wu et al., 2018). Fatigue detection technology is also a potentially effective strategy for monitoring real time fatigue, which was discussed at length in several key academic review papers (Abe et al., 2014; Balkin et al., 2011; Butler and Fee, 2015). However, it was noted that it is critical to evaluate how effective the chosen fatigue-detection technology is, as efficacy can vary. Validity, reliability, sensitivity, specificity, and generalisability must all be considered – in addition to user acceptability (Balkin et al., 2011). Other effective proactive risk controls covered within the literature include alertness promoting strategies such as alerting substances (e.g., caffeine) and on-shift napping (Caldwell et al., 2008; Caruso and Hitchcock, 2010; Sallinen et al., 2017).

#### 3.2.3. Reactive FRMS controls

Based on the documents included in this review, reactive processes were described as being designed to identify the contribution of fatigue to safety reports and events, and include fatigue occurrence reporting, investigations, training and communication. Our review retrieved only one study on fatigue reporting systems (Houston et al., 2012). This study found that fatigue reporting was able to identify otherwise unknown fatigue hazards, and also increased worker feedback (Houston et al., 2012). Incident investigation, and associated data collection and analysis, were also identified as important for managing fatigue-related risk in two other key documents (Ross, 2008; Shaw et al., 2007). FRMS incident investigation was described as focusing on determining systematic causes for errors, rather than placing blame on individual employees (Lerman et al., 2012). This information can be used to identify periods where fatigue-related incidents are more likely (i.e. trends), which can then inform future rostering decisions and control measure implementation (Waclawski and Noone, 2017). Furthermore, positive safety and fatigue outcomes have resulted from alertness promotion/fatigue management training (Barger et al., 2012; Butlewski et al., 2018; Butterfield, 2016; Hauck, 2010; Taylor et al., 2016).

### 3.3. Distribution of FRMS components

**Fig. 3.**
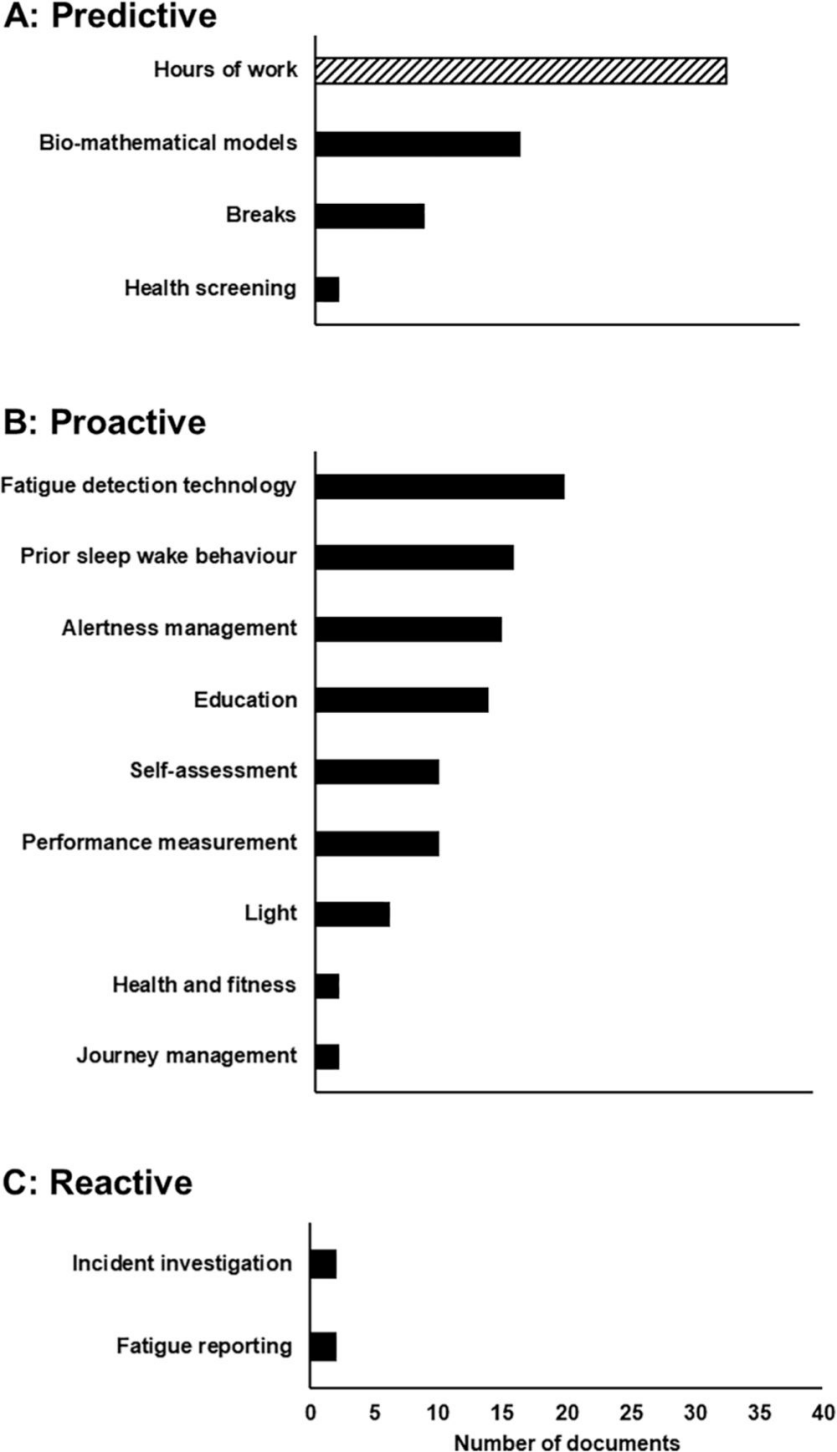
Number of documents addressing FRMS components. Note that some documents addressed more than one component. Note that hours of work is both a predictive measure and also a prescriptive strategy for managing fatigue, rather than part of a risk-based (FRMS) approach.

Each document categorized as evaluating either one or more FRMS component (though not FRMS as a whole) is included in Fig. 3, which demonstrates the spread of FRMS components addressed by the literature.

Panels A, B, and C of Fig. 3 demonstrate the number of documents included in the current review that address predictive, proactive, and reactive FRMS components. This graphical representation suggests that more documents addressing predictive and proactive measures are available. Additionally, the primary areas that have received attention thus far include hours of work, bio-mathematical modelling, fatigue detection technology, prior sleep wake behaviour, alertness management, and education.

As can also be seen in Fig. 3, a number of documents included in this review address hours of work as a strategy for managing fatigue. Hours of work fatigue management strategies can be considered both a predictive control and a prescriptive approach to fatigue management. Of the 34 documents that address hours of work systems for managing fatigue-related risk, nine consist of scientific peer reviewed documents designed to evaluate the impact of certain work schedules and sleep patterns on work and performance outcomes. A further 14 of these documents present key descriptive information on the prescriptive fatigue management systems (i.e., hours of work policies) in place within a variety of industries (e.g., healthcare, police, aviation, transport). On the whole, these studies present compelling evidence that restricting hours of work is likely to limit fatigue to some degree (as a result of greater sleep opportunities and/or reduced time on task). However, none of these documents present a comparison between prescriptive and risk-based systems.

## 4. Discussion

### 4.1. FRMS effectiveness

This review suggests that there is a trend away from prescriptive, compliance-based fatigue management systems - towards flexible risk-based approaches to fatigue management that form the foundation of FRMS (see Fig. 2) (Fourie et al., 2010; Gander, 2015; Gander et al., 2011b; Grech, 2016b). It appears that FRMS are increasingly the preferred option for managing fatigue in large organizations engaged in promoting a flexible safety culture (Waclawski and Noone, 2017). This change can primarily be seen in countries such as Australia, Canada, and the United States (Caldwell et al., 2019), while other areas (e.g., Europe) continue to follow prescriptive fatigue management guidelines (European Working Time Directive (Directive (2003)/88/EC), 2003). It must be noted that there appears to be a downward trend in the number of descriptive documents on FRMS published over recent years (see Fig. 2). This trend has not been accompanied by an increase in the number of documents evaluating FRMS as a whole, suggesting that while FRMS may be established within the literature, uptake and evaluation has not yet occurred widely.

Fatigue management can be categorized on a spectrum – ranging from entirely prescriptive to entirely risk-based systems (i.e., FRMS). Many organisations and industries have implemented components of FRMS within an otherwise prescriptive fatigue management system and fall between these two ends of the spectrum. This tends to include the addition of FRMS-based controls and countermeasures, rather than the adoption of a risk-based framework – i.e., many organisations have chosen certain components of FRMS to implement alongside prescriptive rule sets. In most instances, these FRMS components include specific measures, such as training and education, practical countermeasures, or roster assessment (Barger et al., 2017). Interestingly, the ‘missing’ feature is often the core of FRMS – a risk-based approach to fatigue management. This may be representative of a ‘paradox of the new’ – organisations want evidence of effectiveness but are unwilling to be the first to evaluate the system. As a result, most of the literature in the area is focused on FRMS implementation and components of FRMS, rather than FRMS effectiveness.

The outcomes of FRMS as a whole have been examined in only five studies. The findings of these studies have been summarized in Table 2. In a randomised trial of a fatigue risk management program (similar to FRMS), sleep and alertness were improved in firefighters following implementation of the system (Barger et al., 2017). The program included strategies such as sleep health education, a workplace napping policy, and blackout blinds for sleeping quarters – elements common to FRMS worldwide (Barger et al., 2017). Similarly, sleepiness and fatigue decreased, and situational awareness increased, in a population of nurses following implementation of an FRMS (Dara, 2019). Improved safety outcomes, based on subjective reports, have also been observed in road transport operators as a result of implementation of FRMS (Fourie et al., 2010). Reported outcomes included reduced accidents, improved staff morale, reduced absenteeism and a competitive advantage. Where objective data has been collected within this population (actigraphy) combined with self-report data, implementation of a fatigue management plan (though not an FRMS) has also resulted in improved sleep on duty days, improved performance on a psychomotor vigilance task, and increased fatigue management activities (e.g., education, alertness strategies, healthy sleep, organisational factors) (Smiley et al., 2010). Another study compared the safety of flights that fell under traditional hours of service rules with those that were managed via FRMS (Lamp et al., 2019). Statistical non-inferiority analyses comparing a total of 80 long-haul flights indicated that the flights managed via FRMS were as safe as those managed under standard operating procedures.

The vast majority of the available evidence on FRMS effectiveness targets specific components of the system (e.g., bio-mathematical models, countermeasures, education), rather than FRMS implementation as a whole. The evidence described within the current review suggests that when considered in isolation, FRMS components positively impact fatigue, safety, and performance. Despite a lack of holistic evidence, it appears reasonable to assume that the combination of FRMS components would similarly result in positive outcomes. The combination of safety management system components is similarly effective in addressing hazards other than fatigue (e.g., hazard identification and risk assessment, reporting, and incident investigation) (Thomas, 2012), which suggests that the combination of measures is likely to have a positive net effect. Nevertheless, future research should focus on tracking key safety metrics where full FRMS systems are implemented, from both a scientific and operational perspective (Gander, 2015; Smiley et al., 2010).

### 4.2. What are the key barriers and enablers to effective implementation of a FRMS?

The primary enablers of, and barriers to, the implementation of FRMS are a set of intra-organisational cultural requisites. One key requisite is the establishment of a shared responsibility framework, without which effective FRMS implementation is not possible. In the context of fatigue management, the shared responsibility model describes the need for employees to use their time away from work to obtain sufficient sleep and to present as fit for duty, in addition to complying with relevant policies, procedures, and organisational requirements (Dawson and McCulloch, 2005; Lerman et al., 2012). Operator and manager roles are defined within FRMS and include key responsibilities such as the development of appropriate workplaces, policies, working time arrangements, training, data collection and management systems, and ongoing improvement processes (Lerman et al., 2012). The shared responsibility model is an existing feature of most workplace safety legislative requirements, whether explicit or implicit, and therefore must be the framework underpinning FRMS. FRMS implementation without an established shared responsibility model is therefore a key barrier (Gander, 2015; Gander et al., 2011a; Jones et al., 2003; Lerman et al., 2012; Lillington and McLeod, 2012). Additional barriers to FRMS implementation are potential associated costs (including financial, resource, and time costs), and the possible complexity of a risk-based framework. Specifically, the cost of implementing both FRMS and the associated organisational cultures and frameworks is identified as a potential barrier (Gander et al., 2011b). These barriers were identified within several documents in the present review (Fourie et al., 2010; Gander et al., 2011b), and suggest that for some smaller organisations, a simpler or more limited version of FRMS may be more appropriate (see below for discussion of a hybrid FRMS model). Other barriers identified included difficulty in providing sufficient oversight (Acton, 2014), and obtaining sufficient managerial and executive buy-in (Butler and Bell, 2017).

A key enabler of successful FRMS implementation identified within the literature is a just organisational culture. If workers feel they cannot report fatigue, or fatigue-related errors/incidents without punitive actions or stigma, implementation of FRMS are unlikely to yield positive results (Grech, 2016a; Lerman et al., 2012). Furthermore, it is key for organisations to have sufficient cultural maturity to make organisational changes in response to frequent or significant reports of fatigue. In addition, the organisation must be willing to design the workplace to limit fatigue likelihood and fatigue-related risk. Organisations must be aware that, even under ideal circumstances, the implementation of a just culture is likely to take some time, and starts with leadership and management buy-in (Butler and Bell, 2017). Education and training strategies are also key enablers of FRMS (Acton, 2014; Caruso et al., 2018; Schlesinger, 2017; Williamson et al., 2013). Generally, education within an FRMS involves providing employees with information regarding sleep (i.e., the importance of obtaining sufficient sleep, and strategies for improving their sleep), and practical strategies for improving alertness at work (Acton, 2014; Theron and van Heerden, 2011). Furthermore, education is critical for management and work health and safety professionals, to ensure that work organisation and scheduling practices allow for sufficient rest time for workers. Providing appropriate training has been shown to assist in promoting positive outcomes of FRMS implementation (Acton, 2014; Barger et al., 2012; Chedsey et al., 2007; Gander et al., 2005; Jackson and Reglar, 2011; Theron and van Heerden, 2011) and is therefore a key enabler.

### 4.3. Policy implications

Before undertaking a detailed discussion around regulatory options for implementing formal FRMS policies and procedures, it is important to ensure that the underpinning regulation and/or legislation is appropriate. Our review of the literature indicates that FRMS are predicated upon key regulatory and legislative requirements. Without these it becomes difficult to implement a formal policy and procedure for FRMS. We believe it is critical for regulators to enshrine some key concepts in regulation in order to promote the effectiveness of formal FRMS policy.

First, fatigue must be explicitly defined as a hazard under relevant Work Health and Safety (WHS) regulations. Without a formal identification, FRMS policy and procedure would likely need to be dealt with under general provisions of WHS law. As can be seen from FRMS regulatory initiatives in other jurisdictions, it is critical that fatigue be identified. If we are to explicitly define fatigue as a hazard, the regulatory environment should recognize and acknowledge that fatigue cannot be eliminated as a hazard. People who are required to work shift work—especially in the early hours of the morning—will be unavoidably fatigued due to circadian variations in fatigue and alertness irrespective of the duration of recent sleep and wake or the nature of the task undertaken.

It is important that the primary responsibility for employers and employees vis-à-vis fatigue is to *minimise fatigue-related risk* rather than to eliminate fatigue *per se.* This is a very important distinction. While fatigue is to a certain extent unavoidable, it is still possible to reduce fatigue-related risk through the use of mitigations other than changes to the working time arrangement. It is our view that a formal FRMS regulatory initiative should be based on the regulatory requirement to minimise. In simple terms, we are asking organisations to ensure that their staff are working safely even when fatigued. Because changes to the working time arrangements are difficult to effect and often provide minimal changes to the risk profile, a risk-focussed approach will help organisations move away from approaches that consider reduced hours of work as the sole mechanism whereby we can reduce risk.

Second, it is also necessary to establish a ‘*shared responsibility’* model for accountability for identifying and mitigating fatigue-related risk. Irrespective of the hours an individual works, most of the sleep obtained by an individual occurs outside of the workplace. As such, it is difficult to require an employer to monitor fatigue and fitness for duty in employees. Most WHS jurisdictions across the globe define dual duty-of-care obligations. Formal FRMS policies will require a mechanism to ensure and document that:

- employees are given an adequate opportunity to sleep, rest and recover in order to continue working safely; and
- employees *use the allocated sleep opportunity* to rest and recover in order to continue working safely.

Critically, regulation of accountability for managing fatigue-related risk will also need to define employee obligations explicitly under WHS law. We suggest that (a) and (b) above are supplemented by a reciprocal obligation:

- employees are required to notify their organisation if they have failed to obtain sufficient sleep in order to work safely.

It is important to note that (c) will require the development of guidance materials that indicate the threshold durations of sleep and/or wake needed to work safely or, conversely, to be ‘deemed impaired’ (Dawson et al., 2020).

Without the enabling regulation outlined above, it may be difficult to implement formal FRMS policies and procedures. If we assume that these three principles can be enacted vis-à-vis fatigue, based on the extant literature, there is little doubt that the potential benefits of a formal FRMS are significant in terms of better workplace safety *and* increased operational flexibility. There is now consistent emerging evidence from a range of industries and jurisdictions indicating the likely benefits of FRMS. This is hardly surprising since FRMS is merely a hazard specific operationalisation of broader safety trends over the last few decades. That is, the shift away from prescriptive regulation to performance-based regulation initiated by Lord Robens in the 1970's and the subsequent development of Risk and Safety Management Systems frameworks in the late 20th and early 21st centuries.

However, it is still early days. Definitive evidence will likely take another 5–10 years of formal evaluation studies in order to work out both the optimum regulatory framework(s) and the influence of organisational culture on the implementation and success of a formal FRMS. Until we have such data, most jurisdictions have cautiously adopted formal FRMS principles and limited their use to industries that have the maturity and infrastructure to implement and monitor less prescriptive approaches.

It is also worth noting that in many industries there is already a very strong *informal* FRMS operating. Employees who have had to work regularly while fatigued have often developed very sophisticated and effective ways of mitigating risk in order to continue to work safely (Dawson et al., 2012; Dawson et al., 2015). The regulatory transition to a less prescriptive approaches provides a significant opportunity for organisations to acknowledge and incorporate much of the good informal practice as a central element of the formal FRMS.

It is our view that the success (or otherwise) of a formal FRMS is to a certain extent, inevitably predicated on the broader cultural maturity and pre-existing safety infrastructure of the organisation adopting it. Notwithstanding the possibility that a formal FRMS can provide the impetus for organisations to move toward a comprehensive Safety Management Systems (SMS) framework. FRMS typically assume organisations have a clear understanding of the foundational principles of SMS (e.g., ISO 45000 or local equivalent) and Risk Management (e.g., ISO 31000 or local equivalent). If FRMS regulation is to be introduced, these broader principles should also be considered a key enabler for effective FRMS regulation.

In our opinion, it is essential that regulators reflect on both the significant potential benefits of a formal FRMS, and the potential risk(s) associated with introducing a formal FRMS regulatory approach. Based on the published data, the key regulatory risks are as follows:

- smaller, less well-resourced companies may not be able to ensure the broader SMS/risk management context required to underpin a formal FRMS and this could inadvertently result in inadvertent *de facto* deregulation (i.e., the potential for extended work hours without associated fatigue management strategies);
- regulators may not have a sufficiently well-trained or well-resourced inspectorates to ensure compliance with sufficient rigour to discourage rational ‘cheating’;
- the costs of introducing and maintaining a FRMS may outweigh the benefits especially for organisations or work groups for whom fatigue-related risk might be quite low;
- there are still many industries and/or jurisdictions where the empirical evidence supporting the use of FRMS is not yet definitive; and
- in some industries and jurisdictions, non-prescriptive approaches to regulation are considered inappropriate and difficult to realise due to cultural and political beliefs around the role of government.

Given the above, few jurisdictions have chosen to move quickly from a traditional prescriptive approach to a pure performance-based regulatory framework for managing fatigue-related risk. Indeed, those that have, often find that it was not as successful as had been hoped (McCulloch et al., 2003). Organisations required to develop a formal FRMS while appreciative of the increased operational flexibility often complained about a lack of regulatory guidance supporting the preparation of a formal FRMS, the expense of developing a formal FRMS policy and the inconsistent regulatory response by inspectorates.

It must be noted that while the evidence appears to support the use of FRMS (though future evaluation is required – as discussed above), full FRMS implementation may not be appropriate for all organisations and industries. Where organisations have existing SMS supported by a mature safety culture, in addition to appropriate resources (both financial and personnel), FRMS implementation is likely to be a smooth process. Such organisations are likely to be larger in scale and with existing infrastructure available to support such a transition. Within this context, FRMS implementation could occur on a short timescale based on regulatory advice or requirements.

Conversely, for smaller organisations – or those with immature safety cultures and/or limited resources – the implementation of a full FRMS may be out of reach in the near future. These organisations may also find that, due to cultural concerns, workers and management are not immediately willing to engage with a risk-based approach to fatigue management under the shared responsibility model. Furthermore, the development of a tailored, risk-based fatigue management system may require more resources than the organisation wants or needs (i.e., if they can operate comfortably under prescriptive systems and do not require additional flexibility).

Not surprisingly, most regulators have opted to adopt a transitional approach in which the advantages of FRMS are maximised and disadvantages are minimised. This has typically been achieved through the use of *hybridised* regulatory models (e.g., current United States Federal Aviation Administration regulations for commercial pilots, and Australian heavy vehicle fatigue management). These models typically involve a multi-tiered approach whereby the regulator defines a traditional prescriptive rule-based ‘hours of work’ framework (for organisations who do not require additional flexibility with regard to hours of work) and an alternate compliance/waiver mechanism for those who want to operate outside the traditional prescriptive framework.

In some cases, the provision of the alternate compliance/waiver mechanism permits regulators to ‘tighten’ current prescriptions that are demonstrably unsafe but have been permitted due to political pressure or historical precedent. The political calculus has been that (1) alternate compliance via FRMS provides governments (on behalf of the community) with clear evidence of a safe system of work and (2) provides industry with a clear mechanism for justifying the operational flexibility required can be done safely – as they have claimed.

In regulatory terms, simple prescriptive models typically identify categorical and/or compound rules or dimensions defining one or more of the following:

- minimum ‘time-off-task ‘within a shift (i.e., rest breaks);
- shift maxima;
- minimum breaks between shifts (i.e., recovery breaks);
- aggregate total hours per week/fortnight/month of day and/or night work; and
- minimum breaks between sequences of shifts (i.e., reset breaks).

These dimensions of the working time arrangements are then used to define lower and upper acceptable values for (a) through (e) that define at least three ‘tiers’ of risk. For example, the lower limit for shift duration may be (nominally) set at 8 h and the upper limit at 12 h. Working time arrangements with values below the lower limit are typically considered in the *green* zone. Working time arrangements with values between the lower and upper threshold values are considered in the *yellow/orange* zone and working time arrangements with one or more values above the upper threshold are considered in the *red/black* zone. These can be defined in terms of zones of relative risk, whereby:

- where all values of (a) through (e) within a given time period (e. g., a week or month) fall below specified lower threshold values (e.g., 12 h max shift duration, 10 h break minimum) the working time arrangement is likely to constitute a safe-system-of-work and requires no additional mitigation i.e., it is in the *green* zone.
- where one or more of the lower threshold values for (a) through (e) are exceeded but none of the upper threshold values are exceeded *and* the increased risk can be reasonably offset by appropriate additional risk mitigation i.e., the *yellow/orange* zone.
- where one or more of the upper threshold values for (a) through (e) are exceeded and it is considered unlikely that the additional risk can be sufficiently mitigated to ensure a safe-system-of-work i.e., the *red/black* zone.

**Fig. 4.**
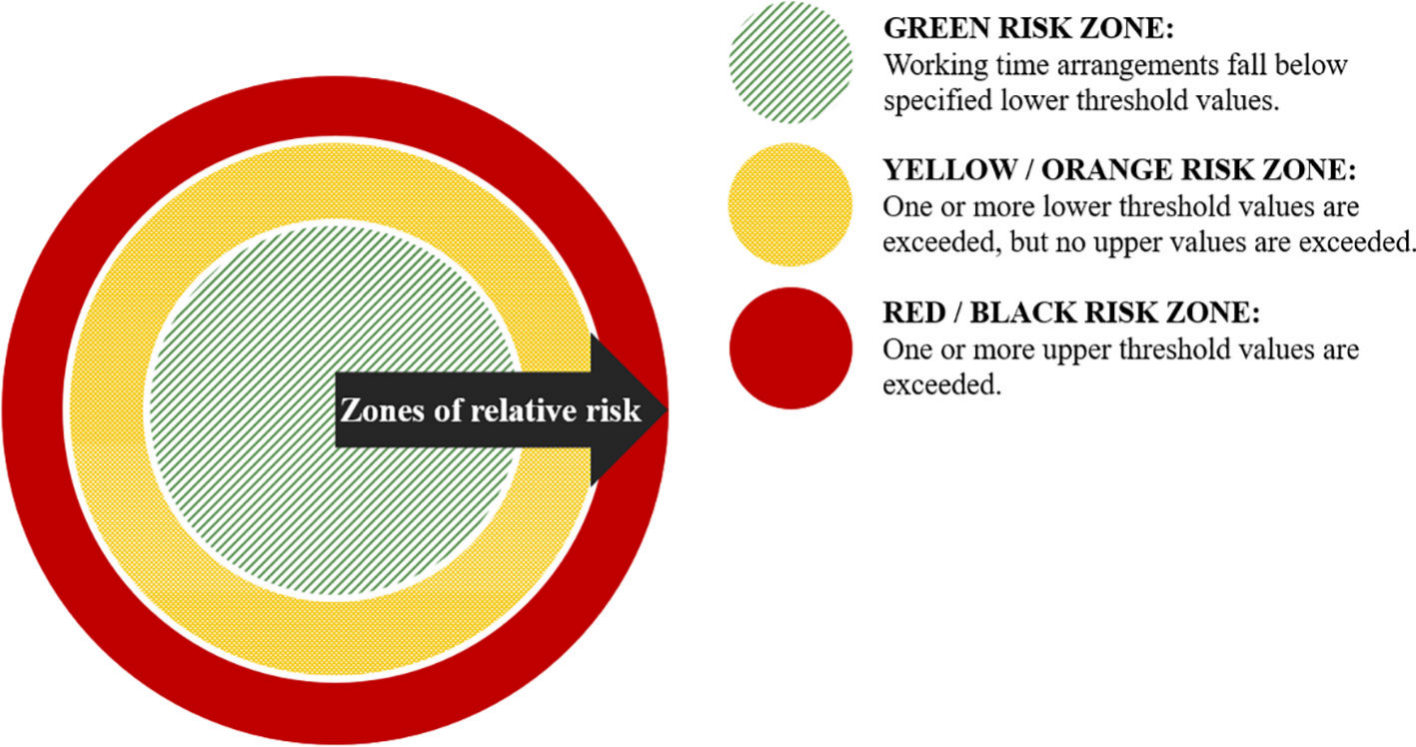
Zones of relative risk. Note that this is a simplified version of the factors that determine whether fatigue risk assessment and additional control measures are required.

Fig. 4 illustrates the concept of relative zones of risk. Where the conditions described in (a), above, are met, and risk can be categorised as being in the ‘green zone’, no additional control measures are required. However, where relative risk is identified as being in the ‘yellow’ or ‘red’ zones, additional fatigue risk assessment and mitigation procedures would be necessary.

### 4.4. Minimum FRMS requirements

This review has highlighted some lack of clarity with respect to what a Fatigue Risk Management System actually entails, and has reinforced that our current approaches to managing fatigue in occupational settings form a spectrum, from purely prescriptive approaches to limiting working hours through multi-faceted fatigue management programs, through to comprehensive FRMS. To this end, there is a need to define the minimum requirements for what constituted a fatigue risk management system. The following could form the basis of the minimum requirements for an FRMS:

1. the adoption of a risk-based approach to fatigue, with multiple mechanisms put in place for the identification of fatigue-related hazards, and the adoption of appropriate controls tailored to each hazard;
2. the adoption of a multi-level approach to hazard identification and risk management that includes predictive, proactive, and reactive processes; and
3. a set of process for the collection of data to assist in hazard identification, and to enable monitoring of the on-going effectiveness of fatigue risk controls.

The review has also highlighted that a comprehensive FRMS aligns closely to the existing components of a Safety Management System (SMS), and wherever possible, an organisation’s FRMS should be integrated into the core elements of an SMS, including hazard identification and risk management, safety occurrence reporting and investigation, and ongoing safety assurance processes. Similarly, experience in industries that have adopted a comprehensive approach to an FRMS has demonstrated the need for strong management commitment, a participatory governance framework, and most importantly embedding fatigue management within a Just Culture framework that supports individuals to make informed decisions about their own fitness for duty on a day-today basis.

### 4.5. Limitations and future directions

While this style of review is effective in identifying relevant studies, reviews, and policy documents, there are some limitations. Primarily, no documents using a randomised controlled trial methodology were identified based on the search criteria. In addition, few documents used methodologies outside of descriptive narratives or policy recommendations. This resulted in an inability to assess bias or collate a body of qualitative or quantitative data for *meta*-analysis. This is likely due to the subject area included within this review – which does not lend itself to traditional pre/post evaluation or comparison.

A critical finding that has come from this review is the lack of studies performed to date designed to evaluate FRMS as a whole (rather than component parts). It is our hope that future research (both academic and within-organisation) will be performed to add to this body of work. A body of evaluative evidence would likely provide organisations, industries, and regulatory bodies with guidance and support for future FRMS implementation.

### 4.6. Conclusions

A review of FRMS literature provides key information on FRMS effectiveness and implementation. Each component of FRMS that has been evaluated has resulted in a reduction of fatigue and/or improved workplace safety outcomes. While minimal data are available on the effectiveness of FRMS as a whole, it would be logical to surmise that combined, FRMS components are likely to improve safety outcomes. Unwillingness of organisations and industries to adopt FRMS in its entirety may stem from this lack of available comprehensive data. This is a paradoxical problem – given that implementation is required before such data can be reported. As current trends indicate the increased use of risk-based fatigue management, it is likely that the relevant data will be available in coming years. At this stage, successful FRMS implementation appears predicated on organizational culture, appropriate training and education on FRMS components, and – critically – commitment from both workers and management under a shared responsibility framework. From a regulatory perspective, the implementation of FRMS may in the first instance be done via a hybrid model, to maximise the potential benefits of FRMS, while minimizing challenges. Under a hybrid model, relatively ‘strict’ hours of work compliance options could be available for organisations who do not have the desire or capacity to implement FRMS. This would ideally be combined with an alternative option for organisations who require increased regulatory flexibility. In this case, an alternative compliance/waiver mechanism could be used, where FRMS implementation – and a safe system of work – can be demonstrated.

## Supplementary Material

Supplemental Material

## Figures and Tables

**Table 1:** Search terms.

Population terms (OR)	AND	Intervention terms (OR)

Work Health and Safety		Fatigue management
Health		Fatigue risk management system
Wellbeing		Fatigue management plan
Safety		Fatigue management program
Injury		Fatigue risk
Accident		Fatigue hazard
Risk		

**Table 2:** Data extraction from documents that included FRMS/FMP evaluation.

Document	Location	Population	Study details	Was a quantitative risk assessment component included?	Peer reviewed?	Outcomes
Barger et al., 2017	Australia	Firefighters (n > 500)	Randomised trial of 34 fire stations. Half allocated to Fatigue Risk Management Program (FRMP). FRMP included • Sleep health education • Sleep disorder screening • Black out shades • Napping policy Baseline and follow up surveys (one year later)	Not specified	No (conference abstract)	• 81% of participants reported black out shades improved their sleep • 79% used napping policy • FRMP group reported significantly improved sleep p < 0.001 • 49% of FRMP group reported increased sleep duration, compared with 23% in control group • FRMP group reported being less sleepy in follow up survey p < 0.001 • Participants in FRMP group reported that the program was “important and helpful”
Dara, 2019	New Zealand	Surgical intensive care unit nurses (n = 36)	Customised performance-based FRMS implemented. Pre-FRMS, FRMS, and post-FRMS assessment of fatigue and situational awareness. • Self-reported fatigue and sleepiness • Actigraphy • Vigilance testing • Situational awareness estimation	Not specified	No (conference abstract)	• Overall decrease in fatigue and sleepiness scores in post-FRMS phase compared with pre-FRMS • Overall increase in situational awareness in post-FRMS phase compared with pre-FRMS
Fourie et al., 2010	Primarily Australia and New Zealand	Transport industry workers (regulatory bodies, transport managers, researchers, other). Organisations: n = 12 Individuals: n = 59	Interviews conducted to obtain perspectives on FRMS implementation, including advantages and disadvantages.	Yes	No (industry conference paper)	• Improved safety (reported by n = 6 organisations) • Improved staff morale (n = 4) • Reduced absenteeism (n = 2) • Competitive advantage (n = 2) • Advanced compliance (n = 2) • Change in regulatory approach (n = 3) • Perceived potential for abuse (n = 2)
Smiley et al., 2010	Canada	Drivers aged 24 – 64 years (n = 77)	Self-report (mood, fatigue, sleep), performance (psychomotor vigilance task (PVT)) and actigraphy data collected pre- and post-Fatigue Management Plan (FMP) implementation. 8 – 10 day data collection each time. FRMS included: • Education on fatigue and sleep disorders • In home screening and CPAP treatment for sleep disordered breathing • Examination of corporate culture • Assessment of scheduling practices Organisational evaluation also performed (Alertness Management Strategies Evaluation (AMSE) questionnaire). Focus groups regarding scheduling practices.	No	No (industry report)	• Improved sleep duration and quality post-FMP compared with pre-FMP • Reduction in critical events (microsleeps, near misses) • Improved PVT performance on rest days for participants treated for sleep apnea • Significant increases in reported and perceived fatigue management activities post-implementation. • No scheduling improvements reported • Fewer road infractions and accidents • Reduced absent days per kilometer travelled
Lamp et al., 2019	United States	Pilots (n = 40)	80 long-haul flights between the United States and Taiwan, and the United States and Australia. Comparison of safety outcomes on flights managed using Safety Standard Operation (SSO) and Alternative Method of Compliance (AMOC). AMOC flight exceeded standard flight time limitations and employed FRMS strategies. Safety performance indicators (SPIs) assessed as outcome measures: • In-flight sleep • Cognitive performance • Self-reported fatigue • Self-reported sleepiness	Yes	Yes (published in Accident Analysis and Prevention journal)	• AMOC flight found to be as safe as SSO flight (non-inferior) on SPIs.

**Table 3:** FRMS/FMP evaluation categories.

Type of evaluation provided within the document
Descriptive of FRMS
Descriptive of FRMS within a particular organization/industry
Evaluation of one FRMS component
Evaluation of more than one FRMS component
Evaluation of FRMS/FMP as a whole
