# Supplementary material for: How effective are Fatigue Risk Management Systems (FRMS)? A review
Source: Accid Anal Prev. Author manuscript; Available in PMC 2023 Feb 1. (PMC8806333; doi:10.1016/j.aap.2021.106398)
Supplement: Supplemental Material [file NIHMS1769244-supplement-Supplemental_Material.docx]

Supplementary Materials

1. Search procedure
   1. Initial search strategy

This process initially sought to achieve a highly comprehensive set of potential ‘interventions’ that can be found within a Fatigue Risk Management System (FRMS). The initial assumptions of the research team were that relatively few papers exist in the academic literature that relate specifically to FRMS as a whole, and that significantly more papers pertain to individual workplace ‘interventions’ to manage fatigue outside the broader context of an FRMS.

Within the search framework, the ‘interventions’ that were assessed were defined in terms of: 1) a specific hazard identification process (such as bio-mathematical modelling of rosters); 2) a specific risk assessment process (such as task analysis or incident investigation); or 3) a specific control of fatigue-related risk (such as provision of napping policy and facilities).

Each of these ‘interventions’ are fundamental elements of an FRMS in industries which use irregular working hours[1, 2]. The initial aim was to define a list of potential search terms for each of the population (used with the Boolean OR operator) and intervention (used with the Boolean OR operator) elements and use the AND operator to search for papers where one or more of the desired populations and desired interventions were both present.

To this end, the search terms listed on the following page were trialed for sensitivity and specificity.

**Supplementary Figure 1.** Initial search terms

1.2 Initial search strategy outcomes

Preliminary searches using the collaboratively developed algorithms very quickly demonstrated that this approach exhibited:

***Over inclusive:*** The approach of trying to develop a *comprehensive* search algorithm that was sensitive enough to identify all potential *populations* associated with a Fatigue Risk Management System (FRMS) yielded many millions of potential papers, as was the case with attempting to develop a *comprehensive* search algorithm that was sensitive enough to identify all potential *interventions* associated with a Fatigue Risk Management System (FRMS)

***Poor-specificity:*** The approach of trying to achieve *specificity* through using the AND operator to find only papers where both populations *and* interventions relevant to FRMS were represented was insufficient to counter the effects of hyper-sensitivity, and only reduced the *tens of millions* of hits to *millions* of hits.

In short, the *over inclusivity* and *poor specificity* in the preliminary search terms is to a large degree a factor of the large number of potential search terms that yield some results relating to the desired subject matter, but also a large number of non-related “hits”. For instance, terms such as “procedure” or “management” are too generic by way of terminology to assist in this type of systematic review.

This is exacerbated by the fact that the term “fatigue” alone is more frequently used in the engineering sciences (component/construction/materials/concrete/metals/composites) than in relation to human fatigue. Attempts at reducing hyper-sensitivity of the preliminary search algorithms by way of excluding this research was found to be nigh on impossible without significant impacts on specificity.

1.3 Evaluating risk of bias and quality assessment

All peer-reviewed literature selected for analysis in the systematic review was intended to be subjected to an evaluation of risk of bias using the Cochrane Collaboration’s tool for assessing risk of bias 4 [3]. Additionally, quality assessments are typically used within systematic reviews to identify and quantify certain research and reporting elements. This can be done using tools such as the EPHPP Quality Assessment Tool for Quantitative Studies. However, given the nature of the research question, and the focus on a complex organisational management system, it was found that a large proportion of studies would be assessed as high risk of bias and low ratings of quality. This is compared with more traditional research involving clinical interventions where methods such as a randomised controlled trial (RCT) is able to implemented. Such methodological rigour in field studies is nearly impossible in the context of workplace risk-management interventions. Accordingly, higher levels of risk of bias and/or quality assessments were not used to exclude pieces of literature from the systematic review, but are noted as a necessary limitation of the review. However, documents were assessed in relation to the degree of evaluation and evidence provided. Specifically, documents were assigned scores from 1 – 5 from 1 (purely descriptive of FRMS) to 5 (evaluation of FRMS as a whole). Classification can be seen in the document evaluation section below.

1. Full reference list included in review

**Supplementary Table 1**. Documents and associated evaluation scores

**Note**:

1. Descriptive of FRMS
2. Descriptive of FRMS within a particular organization / industry
3. Evaluation of one FRMS component
4. Evaluation of more than one FRMS component
5. Evaluation of FRMS as a whole

| # | Document | Year | Score |
| --- | --- | --- | --- |
| 1 | Abe, T., Mollicone, D., Basner, M., & Dinges, D. F. (2014). Sleepiness and safety: Where biology needs technology. *Sleep and Biological Rhythms*, 12(2), 74-84. | 2014 | 1 |
| 2 | Acton, S. (2014). Developing a fatigue risk management system: Case studies on education and mitigation strategies. SPE Annual technical Conference and Exhibition; Amsterdam, The Netherlands, 27-29 October 2014. | 2014 | 4 |
| 3 | Adams-Guppy, J., & Guppy, A. (2003). Truck driver fatigue risk assessment and management: A multinational survey. *Ergonomics*, 46(8), 763-779. | 2003 | 4 |
| 4 | Allen, P., Wadsworth, E., & Smith, A. (2007). The prevention and management of seafarers' fatigue: a review. *International Maritime Health*, 58(1-4), 167-177. | 2007 | 2 |
| 5 | Anderson, C., Grunstein, R. R., & Rajaratnam, S. M. W. (2013). Hours of work and rest in the rail industry. *Internal Medicine Journal*, 43(6), 717-721. | 2013 | 2 |
| 6 | Arlinghaus, A., Lombardi, D. A., Courtney, T. K., Christiani, D. C., Folkard, S., & Perry, M. J. (2012). The effect of rest breaks on time to injury - A study on work-related ladder-fall injuries in the United States. *Scandinavian Journal of Work, Environment and Health*, 38(6), 560-567. | 2012 | 3 |
| 7 | Arnaldo, R. M., Comendador, F. G., Sanz, L. P., & Sanz, A. R. (2016). Friendly Fatigue Alert Mobile Apps to Help Aviation Workers Prevent, Identify and Manage Alertness and Fatigue. In P. Arezes (Ed.), Advances in Safety Management and Human Factors (Vol. 491, pp. 421-432). | 2016 | 3 |
| 8 | Arnold, P. K., & Hartley, L. R. (2001). Policies and practices of transport companies that promote or hinder the management of driver fatigue. Transportation Research Part F: Traffic Psychology and Behaviour, 4(1), 1-17. doi:10.1016/S1369-8478(01)00010-9 | 2001 | 2 |
| 9 | Heavy Vehicle (Fatigue Management) National Regulation, (2018). | 2018 | 1 |
| 10 | Baas, P. H., Charlton, S. G., & Bastin, G. T. (2000). Survey of New Zealand truck driver fatigue and fitness for duty. *Transportation Research Part F: Traffic Psychology and Behaviour*, 3(4), 185-193. | 2000 | 3 |
| 11 | Balkin, T. J., Horrey, W. J., Graeber, R. C., Czeisler, C. A., & Dinges, D. F. (2011). The challenges and opportunities of technological approaches to fatigue management. *Accident Analysis and Prevention*, 43(2), 565-572. | 2011 | 2 |
| 12 | Banks, J. O., Avers, K. E., Nesthus, T. E., & Hauck, E. L. (2012). A comparative study of international flight attendant fatigue regulations and collective bargaining agreements. *Journal of Air Transport Management*, 19(1), 21-24. | 2012 | 3 |
| 13 | Barger, L. K., Ayas, N. T., Cade, B. E., Cronin, J. W., Rosner, B., Speizer, F. E., & Czeisler, C. A. (2007). Impact of extended-duration shifts on medical errors, adverse events, and attentional failures. *Public Library of Science Medicine*, 3(12), e487. | 2007 | 3 |
| 14 | Barger, L. K., Lockley, S. W., Rajaratnam, S. M. W., & Landrigan, C. P. (2009). Neurobehavioral, health, and safety consequences associated with shift work in safety-sensitive professions. *Current Neurology and Neuroscience Reports*, 9(2), 155-164. | 2009 | 3 |
| 15 | Barger, L. K., O'Brien, C., Sullivan, J., Wang, W., Lockley, S., Qadri, S., . . . Czeisler, C. A. (2017). Fatigue risk management program increases sleep and alertness in firefighters. *Sleep*, 40, A439-A439. | 2017 | 5 |
| 16 | Barger, L. K., Sullivan, J. P., Vincent, A. S., Fiedler, E. R., McKenna, L. M., Flynn-Evans, E. E., . . . Lockley, S. W. (2012). Learning to Live on a Mars Day: Fatigue Countermeasures during the Phoenix Mars Lander Mission. *Sleep*, 35(10), 1423-1435. | 2012 | 4 |
| 17 | Baskin, P. (2019). Fatigue management to reduce performance risk for space exploration. *Sleep*, 42, A82. | 2019 | 3 |
| 18 | Bauerle, T., Dugdale, Z., & Poplin, G. (2018). Mineworker fatigue: a review of what we know and future directions. In Vision, innovation and identity: step change for a sustainable future, 2018 SME annual conference & expo and 91st annual meeting of the SME-MN section, February 25-28, 2018, Minneapolis, Minnesota (pp. 399-406). Red Hook, NY: Curran Associates Inc. | 2018 | 2 |
| 19 | Baulk, S. D., Biggs, S. N., Reid, K. J., van den Heuvel, C. J., & Dawson, D. (2008). Chasing the silver bullet: Measuring driver fatigue using simple and complex tasks. Accident Analysis and Prevention, 40(1), 396-402. | 2008 | 4 |
| 20 | Belenky, G., Lamp, A., Hemp, A., & Zaslona, J. L. (2014). Fatigue in the workplace. In Sleep Deprivation and Disease: Effects on the Body, Brain and Behavior (Vol. 9, pp. 243-268). | 2014 | 1 |
| 21 | Belenky, G., Wu, L. J., & Jackson, M. L. (2011) Occupational sleep medicine. Practice and promise. In: Vol. 190. Progress in Brain Research (pp. 189-203). | 2011 | 1 |
| 22 | Belval, E. J., Calkin, D. E., Wei, Y., Stonesifer, C. S., Thompson, M. P., & Masarie, A. (2018). Examining dispatching practices for Interagency Hotshot Crews to reduce seasonal travel distance and manage fatigue. *International Journal of Wildland Fire*, 27(9), 569-580. | 2018 | 3 |
| 23 | Berneking, M., Rosen, I. M., Kirsch, D. B., Chervin, R. D., Carden, K. A., Ramar, K., . . . American Academy of Sleep Medicine Board of, D. (2018). The Risk of Fatigue and Sleepiness in the Ridesharing Industry: An American Academy of Sleep Medicine Position Statement. Journal of clinical sleep medicine : JCSM : official publication of the American Academy of Sleep Medicine, 14(4), 683-685. | 2018 | 2 |
| 24 | Boivin, D. & Boudreau, P. (2014). Impacts of shift work on sleep and circadian rhythms. *Pathologie Biologie*, 62(5), 292-301. | 2014 | 1 |
| 25 | Boivin, D. B., Boudreau, P., James, F. O., & Kin, N. (2012). Photic resetting in night-shift work: impact on nurses' sleep. *Chronobiology International,* 29(5), 619-628. | 2012 | 3 |
| 26 | Boivin, D. B., & James, F. (2002). Circadian adaptation to night-shift work by judicious light and darkness exposure. *Journal of Biological Rhythms*, 17(6), 556-567. | 2002 | 3 |
| 27 | Boudreau, P., Dumont, G. A., & Boivin, D. (2013). Circadian adaptation to night shift work influences sleep, performance, mood and the autonomic modulation of the heart. *PloS One*, 8(7). | 2013 | 3 |
| 28 | Bowden, Z. E., & Ragsdale, C. T. (2018). The truck driver scheduling problem with fatigue monitoring. *Decision Support Systems*, 110, 20-31. | 2018 | 3 |
| 29 | Brown, I. D. (1994). Driver Fatigue. *Human Factors*, 36(2), 298-314. | 1994 | 2 |
| 30 | Brown, L., Schoutens, A. M. C., Whitehurst, G., Booker, T., Davis, T., Losinski, S., & Diehl, R. (2014). The effects of light exposure on flight crew alertness levels to enhance fatigue risk management predication models. Probabilistic Safety Assessment and Management PSAM 12. Honolulu, Hawaii | 2014 | 3 |
| 31 | Bustos, D., Guedes, J. C., Alvares, M., Baptista, J. S., Vaz, M., & Torres Costa, J. C. (2019) Real time fatigue assessment: Identification and continuous tracing of fatigue using a physiological assessment algorithm. In: Vol. 202. Studies in Systems, Decision and Control (pp. 257-265). | 2019 | 3 |
| 32 | Butler, C., & Bell, J. (2017). Worker fatigue risk management in practice: Benefits and challenges. *Hazards 27*, Symposium Series 162. | 2017 | 1 |
| 33 | Butler, P., & Fee, W. (2015). Fatigue and the use of wearable technology. SPE E&P Health, Safety, Security and Environmental Conference-Americas, Denver, Colorado, USA. | 2015 | 3 |
| 34 | Butlewski, M., Dahlke, G., Drzewiecka-Dahlke, M., Gorny, A., & Pacholski, L. (2018). Implementation of TPM Methodology in Worker Fatigue Management - A Macroergonomic Approach. In R. H. M. Goossens (Ed.), Advances in Social & Occupational Ergonomics, Ahfe 2017 (Vol. 605, pp. 32-41). | 2018 | 3 |
| 35 | Butlewski, M., Dahlke, G., Drzewiecka, M., & Pacholski, L. (2015). Fatigue of Miners as a Key Factor in the Work Safety System. *Procedia Manufacturing*, 3, 4732-4739. | 2015 | 1 |
| 36 | Butterfield, P. (2016). Workers with irregular hours during seasonal work surges: Promoting healthy sleep. *Workplace Health and Safety*, 64(3), 128. | 2016 | 2 |
| 37 | Buysse, D. J. (2018). Evidence-Based Guidelines for Fatigue Risk Management in Emergency Medical Services: A Step in the Right Direction Toward Better Sleep Health. *Prehospital Emergency Care*, 22, 3-5. | 2018 | 2 |
| 38 | Cabon, P., Deharvengt, S., Berechet, I., Grau, J. Y., Maille, N. P., & Mollard, R. (2011). From flight time limitations to fatigue risk management systems - A way toward resilience. In Resilience Engineering in Practice: A Guidebook (pp. 69-86). | 2011 | 2 |
| 39 | Cabon, P., Deharvengt, S., Grau, J. Y., Maille, N., Berechet, I., & Mollard, R. (2012). Research and guidelines for implementing Fatigue Risk Management Systems for the French regional airlines. *Accident Analysis and Prevention*, 45, 41-44. | 2012 | 2 |
| 40 | Cabon, P., Lancelle, V., Mollard, R., Grau, J. Y., Blatter, C., Kaplan, M., . . . Tirilly, G. (2012). Sleep, fatigue and hours of work of French train drivers. Rail Human Factors around the World. | 2012 | 4 |
| 41 | Caldwell, J. A., Caldwell, J. L., & Schmidt, R. M. (2008). Alertness management strategies for operational contexts. *Sleep Medicine Reviews*, 12(4), 257-273. | 2008 | 1 |
| 42 | Camargo, M., Fox, C., O'Byrne, M., Villarreal, G., & Fraga, K. (2010). Implementing driving fatigue management in oil & gas operations in Brazil. SPE International Conference on Health, Safety and Environment in Oil and Gas Exploration and Production, Rio de Janeiro, Brazil. | 2010 | 4 |
| 43 | Campbell, B. (2018). Preconditions, Regulatory Failure and Corporate Negligence Behind the Lac-Mégantic Disaster. *J Revue générale de droit*, 48, 95-130. | 2018 | 2 |
| 44 | Canadian College of Physicians. (2018). Fatigue risk management toolkit for residents, leaders, and policy makers in Canadian postgraduate medical education. | 2018 | 2 |
| 45 | Caruso, C. C. (2016). Reducing risks to women linked to shift work, long work hours, and related workplace sleep and fatigue issues. *Journal of Women's Health*, 24(10), 789-794. | 2016 | 1 |
| 46 | Caruso, C. C., Baldwin, C. M., Berger, A., Chasens, E. R., Landis, C., Redeker, N. S., . . . Trinkoff, A. (2018). Position statement: reducing fatigue associated with sleep deficiency and work hours in nurses. Nurs Outlook, 65(6), 766-768. | 2018 | 2 |
| 47 | Caruso, C. C., & Hitchcock, E. M. (2010). Strategies for nurses to prevent sleep-related injuries and errors. *Rehabilitation Nursing*, 35(5), 192-197. | 2010 | 2 |
| 48 | Chang, Y. H., Yang, H. H., & Hsu, W. J. (2019). Effects of work shifts on fatigue levels of air traffic controllers. *Journal of Air Transport Management*, 76, 1-9. | 2019 | 4 |
| 49 | Chedsey, G., Kaminski, D., & Kaufmann, J. (2007). Focused oilfield-specific training reduces accident costs by over 10 times. SPE E&P Environmental and Safety Conference, Galveston, Texas. | 2007 | 3 |
| 50 | Chen, C. K., Lin, C., Hou, T. H., Wang, S. H., & Lin, H. M. (2010). A study of operating room scheduling that integrates multiple quantitative and qualitative objectives. *J Nurs Res*, 18(1), 62-74. | 2010 | 3 |
| 51 | Chen, G. X. (2016). Trucker sleep patterns influence safety-critical events. In Atlas Sci (pp. 1-2). WV: Atlas of Science. | 2016 | 3 |
| 52 | Chen, G. X., Fang, Y., Guo, F., & Hanowski, R. J. (2016). The influence of daily sleep patterns of commercial truck drivers on driving performance. Accident Analysis and Prevention, 91, 55-63. | 2016 | 3 |
| 53 | Cheng, Y.-H., & Tian, H.-N. (2019). Train drivers' subjective perceptions of their abilities to perceive and control fatigue. International Journal of Occupational Safety and Ergonomics : JOSE, 1-17. | 2019 | 2 |
| 54 | Cheng, Y. H., Roach, G. D., & Petrilli, R. M. A. (2014). Current and future directions in clinical fatigue management: An update for emergency medicine practitioners. Emergency Medicine Australasia, 26(6), 640-644. doi:10.1111/1742-6723.12319 | 2014 | 2 |
| 55 | Civil Aviation Safety Authority. (2019). Fatigue risk management systems: A step-by-step guide. | 2019 | 2 |
| 56 | Comperatore, C. A., Ng, P. K., & Carvalhais, A. B. (2015) Design Standards Considerations and the Effective Prevention of Operator Fatigue. *Reviews of Human Factors and Ergonomics,* 10, 174-193. | 2015 | 1 |
| 57 | Cumber, E., & Greig, P. R. (2019). Can a tool developed for industry be used to assess fatigue risk in medical rotas? A pilot study of foundation doctors' rotas in a tertiary centre. *BMJ Open*, 9(2). | 2019 | 3 |
| 58 | Dai, J., Luo, M., Hu, W., Ma, J., & Wen, Z. (2018). Developing a fatigue questionnaire for Chinese civil aviation pilots. International Journal of Occupational Safety and Ergonomics. | 2018 | 3 |
| 59 | Dara, S. (2019). Impact of fatigue risk management system on fatigue and situation awareness of surgical intensive care unit nurses. *Internal Medicine Journal*, 49, 19-19. | 2019 | 5 |
| 60 | Darwent, D., Dawson, D., Paterson, J. L., Roach, G. D., & Ferguson, S. A. (2015). Managing fatigue: It really is about sleep. *Accident Analysis and Prevention*, 82, 20-26. | 2015 | 1 |
| 61 | Dawson, D. (2018). Fatigue proofing: the next generation of fatigue risk management. 24^th^ Congress of the European Sleep Research Society, Basel, Switzerland. | 2018 | 1 |
| 62 | Dawson, D., & Bowe, A. (2019). Guidelines for developing and implementing a fatigue management policy in forestry. | 2019 | 2 |
| 63 | Dawson, D., Chapman, J., & Thomas, M. (2012). Fatigue-proofing: a new approach to reducing fatigue-related risk using the principles of error management. *Sleep Medicine Reviews*, 16(2), 167-175. | 2012 | 1 |
| 64 | Dawson, D., Darwent, D., & Roach, G. D. (2017). How should a bio-mathematical model be used within a fatigue risk management system to determine whether or not a working time arrangement is safe? *Accident Analysis and Prevention*, 99, 469-473. | 2017 | 1 |
| 65 | Dawson, D., Ian Noy, Y., Härmä, M., Kerstedt, T., & Belenky, G. (2011). Modelling fatigue and the use of fatigue models in work settings. *Accident Analysis and Prevention*, 43(2), 549-564. | 2011 | 1 |
| 66 | Dawson, D., Mayger, K., Thomas, M. J. W., & Thompson, K. (2015). Fatigue risk management by volunteer fire-fighters: Use of informal strategies to augment formal policy. *Accident Analysis and Prevention*, 84, 92-98. | 2015 | 3 |
| 67 | Dawson, D., & McCulloch, K. (2005). Managing fatigue: It's about sleep. Sleep Medicine Reviews, 9(5), 365-380. | 2005 | 1 |
| 68 | Dawson, D., Reynolds, A. C., Van Dongen, H. P., & Thomas, M. J. (2018). Determining the likelihood that fatigue was present in a road accident: a theoretical review and suggested accident taxonomy. *Sleep Medicine Reviews*, 42, 202-210. | 2018 | 1 |
| 69 | Dawson, D., Searle, A. K., & Paterson, J. L. (2014). Look before you (s)leep: Evaluating the use of fatigue detection technologies within a fatigue risk management system for the road transport industry. *Sleep Medicine Reviews*, 18(2), 141-152. | 2014 | 3 |
| 70 | de Negreiros, A., da Silva, P. R., Arezes, P., Dangelino, R., & Padula, R. S. (2019). Manufacturing assembly serial and cells layouts impact on rest breaks and workers' health. *International Journal of Industrial Ergonomics*, 70, 22-27. | 2019 | 3 |
| 71 | Dingus, T. A., Guo, F., Lee, S., Antin, J. F., Perez, M., Buchanan-King, M., & Hankey, J. (2016). Driver crash risk factors and prevalence evaluation using naturalistic driving data. Proceedings of the National Academy of Sciences of the United States of America, 113(10), 2636-2641. | 2016 | 3 |
| 72 | Dorn, L., Stephen, L., Wåhlberg, A., & Gandolfi, J. (2010). Development and validation of a self-report measure of bus driver behaviour. *Ergonomics*, 53(12), 1420-1433. | 2010 | 3 |
| 73 | Dorrian, J., Baulk, S. D., & Dawson, D. (2011). Work hours, workload, sleep and fatigue in Australian Rail Industry employees. *Applied Ergonomics*, 42(2), 202-209. | 2011 | 4 |
| 74 | Eiter, B. M., Steiner, L., & Kelhart, A. (2014). Application of fatigue management systems: Small mines and low technology solutions. *Mining Engineering*, 66(4), 69-75. | 2014 | 2 |
| 75 | Hours of Service of Railroad Employees; Substantive Regulations for Train Employees Providing Commuter and Intercity Rail Passenger Transportation; Conforming Amendments to Recordkeeping Requirements. Final Rule. Federal Register 76. 50359-50401, (2011a). | 2011 | 2 |
| 76 | Federal Railway Administration (FRA). (2011b). Measurement and Estimation of Sleep in Railroad Worker Employees. Retrieved from US Department of Transportation. | 2011 | 3 |
| 77 | Ferguson, S. A., Lamond, N., Kandelaars, K., Jay, S. M., & Dawson, D. (2008). The impact of short, irregular sleep opportunities at sea on the alertness of marine pilots working extended hours. *Chronobiology International*, 25(2-3), 399-411. | 2008 | 3 |
| 78 | Ferguson, S. A., Smith, B. P., Browne, M., & Rockloff, M. J. (2016). Fatigue in emergency services operations: Assessment of the optimal objective and subjective measures using a simulated wildfire deployment. *International Journal of Environmental Research and Public Health*, 13(2). | 2016 | 4 |
| 79 | Filtness, A. J., & Naweed, A. (2017). Causes, consequences and countermeasures to driver fatigue in the rail industry: The train driver perspective. Applied Ergonomics, 60, 12-21. | 2017 | 4 |
| 80 | Fletcher, A., & Dawson, D. (2001). Field-based validations of a work-related fatigue model based on hours of work. Transportation Research Part F: Traffic Psychology and Behaviour, 4(1), 75-88. | 2001 | 3 |
| 81 | Fletcher, A., Hooper, B., Dunican, I., & Kogi, K. (2015) Fatigue Management in Safety-Critical Operations: History, Terminology, Management System Frameworks, and Industry Challenges. In: Vol. 10. Reviews of Human Factors and Ergonomics (pp. 6-28). | 2015 | 1 |
| 82 | Fletcher, L., Petersson, L., & Zelinsky, A. (2005). Road scene monotony detection in a fatigue management driver assistance system.  Intelligent Vehicles Symposium. | 2005 | 3 |
| 83 | Flower, D., Arnulf, L., Kumarasamy, D., Pelat, F., Phillips, K., Reeves, G., . . . Kostareli, A. (2016). Fatigue in fly in fly out operations. SPE International Conference and Exhibition on Health, Safety, Security, Environment, and Social Responsibility, Stavanger, Norway. | 2016 | 2 |
| 84 | Flynn-Evans, E. E., Arsintescu, L., Gregory, K., Mulligan, J., Nowinski, J., & Feary, M. (2018). Sleep and neurobehavioral performance vary by work start time during non-traditional day shifts. *Sleep Health*, 4(5), 476-484. | 2018 | 3 |
| 85 | Folkard, S., & Lombardi, D. A. (2005). Toward a "Risk Index" to assess work schedules. *Chronobiology International*, 21(6), 1063-1072. | 2005 | 1 |
| 86 | Folkard, S., & Lombardi, D. A. (2007). Modeling the impact of the components of long work hours on injuries and “accidents”. *Am J Ind Med*, 49(11), 953-963. | 2007 | 3 |
| 87 | Folkard, S., Robertson, K. A., & Spencer, M. B. (2007). A Fatigue/Risk Index to assess work schedules. *Somnologie*, 11(3), 177-185. | 2007 | 3 |
| 88 | Fourie, C., Holmes, A., Hilditch, C., Bourgeois-Bougrine, S., & Jackson, P. (2010). What can we learn from small operators that have implemented fatigue risk management systems (FRMS)? Paper presented at the 55th Annual CASS. FSF and NBAA., Tucson, Arizona. | 2010 | 5 |
| 89 | Fournier, P. S., Montreuil, S., & Brun, J. P. (2007). Fatigue management by truck drivers in real life situations: some suggestions to improve training. *Work*, 29(3), 213-224. | 2007 | 2 |
| 90 | Friedl, K. E., Mallis, M. M., Ahlers, S. T., Popkin, S. M., & Larkin, W. (2004). Research requirements for operational decision-making using models of fatigue and performance. *Aviation Space and Environmental Medicine*, 75(3), A192-A199. | 2004 | 1 |
| 91 | Gander, P. (2001). Fatigue management in air traffic control: The New Zealand approach. Transportation Research Part F: Traffic Psychology and Behaviour, 4(1), 49-62. | 2001 | 2 |
| 92 | Gander, P., Graeber, R. C., & Belenky, G. (2010). Fatigue Risk Management. In Principles and Practice of Sleep Medicine: Fifth Edition (pp. 760-768). | 2010 | 1 |
| 93 | Gander, P., Hartley, L., Powell, D., Cabon, P., Hitchcock, E., Mills, A., & Popkin, S. (2011). Fatigue risk management: Organizational factors at the regulatory and industry/company level. Accident Analysis and Prevention, 43(2), 573-590. | 2011 | 1 |
| 94 | Gander, P., Mangie, J., Phillips, A., Santos-Fernandez, E., & Wu, L. J. (2018). Monitoring the Effectiveness of Fatigue Risk Management: A Survey of Pilots' Concerns. Aerospace Medicine and Human Performance, 89(10), 889-895. doi:10.3357/amhp.5136.2018 | 2018 | 4 |
| 95 | Gander, P., Mangie, J., Wu, L., van den Berg, M., Signal, L., & Phillips, A. (2017). Preparing Safety Cases for Operating Outside Prescriptive Fatigue Risk Management Regulations. Aerospace Medicine and Human Performance, 88(7), 688-696. | 2017 | 1 |
| 96 | Gander, P., O'Keeffe, K., Santos-Fernandez, E., Huntington, A., Walker, L., & Willis, J. (2019). Fatigue and nurses’ work patterns: An online questionnaire survey. *International Journal of Nursing Studies*, 98, 67-74. | 2019 | 3 |
| 97 | Gander, P., Purnell, H., Garden, A., & Woodward, A. (2007). Work patterns and fatigue-related risk among junior doctors. *Occupational and Environmental Medicine*, 64(11), 733-738. | 2007 | 3 |
| 98 | Gander, P., van den Berg, M., & Signal, L. (2008). Sleep and sleepiness of fisherman on rotating schedules. Chronobiology International, 25(2-3), 389-398. doi:10.1080/07420520802106728 | 2008 | 3 |
| 99 | Gander, P. H. (2015) Evolving Regulatory Approaches for Managing Fatigue Risk in Transport Operations. In: Vol. 10. Reviews of Human Factors and Ergonomics (pp. 253-271). | 2015 | 2 |
| 100 | Gander, P. H., Mangie, J., van den Berg, M. J., Smith, A. A. T., Mulrine, H. M., & Signal, T. L. (2014). Crew Fatigue Safety Performance Indicators for Fatigue Risk Management Systems. Aviation Space and Environmental Medicine, 85(2), 139-147. | 2014 | 3 |
| 101 | Gander, P. H., Marshall, N. S., Bolger, W., & Girling, I. (2005). An evaluation of driver training as a fatigue countermeasure. Transportation Research Part F: Traffic Psychology and Behaviour, 8(1), 47-58. | 2005 | 3 |
| 102 | Geiger-Brown, J. M., Lee, C. J., & Trinkoff, A. M. (2012). The role of work schedules in occupational health and safety. In R. J. Gatchel & I. Z. Schultz (Eds.), Handbook of occupational health and wellness. (pp. 297-322). New York, NY: Springer Science + Business Media. | 2012 | 3 |
| 103 | Gilbreth, L. (2016). Fatigue as it Affects Nursing. *The American Journal of Nursing*, 116(8), 66-69. | 2016 | 2 |
| 104 | Goldenhar, L. M., Hecker, S., Moir, S., & Rosecrance, J. C. (2003). The “goldilocks model” of overtime in construction: not too much, not too little, but just right. Journal of Safety Research, 34(2), 215-226. | 2003 | 3 |
| 105 | Grace, R., & Benjamin, A. L. (1999). Application of a heavy vehicle drowsy driver detection system. doi:10.4271/1999-01-3754 | 1999 | 3 |
| 106 | Grech, M. R. (2016). Fatigue Risk Management: A Maritime Framework. *International Journal of Environmental Research and Public Health*, 13(2). | 2016 | 2 |
| 107 | Grogan, E. L., Stiles, R. A., France, D. J., Speroff, T., Morris, J. A., Nixon, B., . . . Pinson, C. W. (2004). The impact of aviation-based teamwork training on the attitudes of health-care professionals. *Journal of the American College of Surgeons*, 199(6), 843-848. | 2004 | 3 |
| 108 | Gurubhagavatula, I. (2013). Does the rubber meet the road? Addressing sleep apnea in commercial truck drivers. In Sleep 2012 Nov; 35(11):1443-1444. PA: Sleep. | 2013 | 3 |
| 109 | Guttkuhn, R., Trutschel, U., Heitmann, A., Aguirre, A., & Moore-Ede, M. (2003). Human fatigue risk simulations in 24/7 operations. | 2003 | 3 |
| 110 | Haluza, D., Schmidt, V. M., & Blasche, G. (2019). Time course of recovery after two successive night shifts: A diary study among Austrian nurses. *Journal of Nursing Management*, 27(1), 190-196. | 2019 | 3 |
| 111 | Hansen, J. H., Geving, I. H., & Reinertsen, R. E. (2010). Adaptation rate of 6-sulfatoxymelatonin and cognitive performance in offshore fleet shift workers: a field study. *Int Arch Occup Environ Health*, 83(6), 607-615. | 2010 | 3 |
| 112 | Hauck, E. L. (2011). Development and evaluation of a fatigue countermeasure training program for shiftworkers. (72). ProQuest Information & Learning | 2011 | 3 |
| 113 | Haynes, A. L. (2017). Reduction of Sleep Deprivation and Fatigue in Mass Transit Rail Operators. *Workplace Health and Safety*, 65(8), 333-336. | 2017 | 2 |
| 114 | Heinzmann, J., Tate, D., & Scott, R. (2008). Using technology to eliminate drowsy driving. SPE International Conference on Health, Safety, and Environment in Oil and Gas Exploration and Production, Nice, France. | 2008 | 3 |
| 115 | Hellerström, D., Eriksson, H., Romig, E., & Klemets, T. (2010). Flight time limitations and fatigue risk management: A comparison of three regulatory approaches. | 2010 | 4 |
| 116 | Honn, K. A., van Dongen, H. P. A., & Dawson, D. (2019). Working time society consensus statements: Prescriptive rule sets and risk management-based approaches for the management of fatigue-related risk in working time arrangements. *Industrial Health*, 57(2), 264-280. | 2019 | 1 |
| 117 | Houston, S., Dawson, K., & Butler, S. (2012). Fatigue reporting among aircrew: Incidence rate and primary causes. *Aviation Space and Environmental Medicine*, 83(8), 800-804. | 2012 | 3 |
| 118 | IATA, ICAO, & IFAPLA. (2011). Fatigue Risk Management Systems - Implementation Guide for Operators. Montreal, Canada: IATA. | 2011 | 1 |
| 119 | Ingre, M., Van Leeuwen, W., Klemets, T., Ullvetter, C., Hough, S., Kecklund, G., . . . Åkerstedt, T. (2014). Validating and extending the three process model of alertness in airline operations. PLoS ONE, 9(10). | 2014 | 3 |
| 120 | International Air Transport Association. (2014). Fatigue Safety Performance Indicators (SPIs): A Key Component of Proactive Fatigue Hazard Identification. Montreal, Canada: IATA. | 2014 | 4 |
| 121 | International Civil Aviation Organization. (2016). DOC 9966: Manual for the Oversight of Fatigue Management Approaches. Version 2 (revised). | 2016 | 2 |
| 122 | Jackson, J. E., Sanquist, T., Campbell, J., Lee, E. B., & Van Dongen, H. P. A. (2013). Fatigue in Highway Construction Workers Risks and Countermeasures in Rapid Renewal Project Schedules. *Transportation Research Record* (2347), 11-18. | 2013 | 3 |
| 123 | Jackson, M. L., Banks, S., & Belenky, G. (2014). Investigation of the effectiveness of a split sleep schedule in sustaining sleep and maintaining performance. *Chronobiology International*, 31(10), 1218-1230. | 2014 | 3 |
| 124 | Jackson, P. G., & Reglar, J. M. (2011). Developing online fatigue awareness and countermeasures training for a distributed workforce. SPE European Health, Safety and Environmental Conference in Oil and Gas Exploration and Production, Vienna, Austria | 2011 | 3 |
| 125 | James, F. O., Waggoner, L. B., Weiss, P. M., Patterson, P. D., Higgins, J. S., Lang, E. S., & Van Dongen, H. P. A. (2018). Does Implementation of Biomathematical Models Mitigate Fatigue and Fatigue-related Risks in Emergency Medical Services Operations? A Systematic Review. *Prehospital Emergency Care*, 22, 69-80. | 2018 | 2 |
| 126 | James, F. O., Walker, C. D., & Boivin, D. B. J. C. i. (2004). Controlled exposure to light and darkness realigns the salivary cortisol rhythm in night shift workers. *Chronobiology International*, 21(6), 961-972. | 2004 | 3 |
| 127 | James, L., Samuels, C. H., & Vincent, F. (2018). Evaluating the Effectiveness of Fatigue Management Training to Improve Police Sleep Health and Wellness: A Pilot Study. *Journal of Occupational and Environmental Medicine*, 60(1), 77-82. | 2018 | 3 |
| 128 | Jensen, A., & Dahl, S. (2009). Truck drivers hours-of-service regulations and occupational health. *Work-a Journal of Prevention Assessment & Rehabilitation*, 33(3), 363-368. | 2009 | 3 |
| 129 | Jepsen, J. R., Zhao, Z., Pekcan, C., Barnett, M., & Van Leeuwen, W. M. A. (2017). Risk factors for fatigue in shipping, the consequences for seafarers’ health and options for preventive intervention. In Maritime Psychology: Research in Organizational and Health Behavior at Sea (pp. 127-150). | 2017 | 2 |
| 130 | Jones, C., Dorrian, J., & Dawson, D. (2003). Legal Implications of Fatigue in the Australian Transportation Industries. *Journal of Industrial Relations*, 45(3), 344-359. | 2003 | 2 |
| 131 | - Karlen, W., Cardin, S., Thalmann, D., & Floreano, D. (2010). Enhancing pilot performance with a SymBodic system. Annual International Conference of the IEEE Engineering in Medicine and Biology Society. | 2010 | 3 |
| 132 | Knecht, C. P., Muehlethaler, C. M., & Elfering, A. (2016). Nontechnical skills training in air traffic management including computer-based simulation methods: From scientific analyses to prototype training. *Aviation Psychology and Applied Human Factors*, 6(2), 91-100. | 2016 | 3 |
| 133 | Kosmadopoulos, A., Sargent, C., Zhou, X., Darwent, D., Matthews, R. W., Dawson, D., & Roach, G. D. (2017). The efficacy of objective and subjective predictors of driving performance during sleep restriction and circadian misalignment. *Accident Analysis and Prevention*, 99, 445-451. | 2017 | 4 |
| 134 | Lamp, A., Chen, J. M. C., McCullough, D., & Belenky, G. (2019). Equal to or better than: The application of statistical non-inferiority to fatigue risk management. *Accident Analysis and Prevention*, 126, 184-190. | 2019 | 5 |
| 135 | Lehrer, A. M. (2015) A Systems-Based Framework to Measure, Predict, and Manage Fatigue. In: Vol. 10. Reviews of Human Factors and Ergonomics (pp. 194-252). | 2015 | 1 |
| 136 | Leonard, B., Jerdack, N., Magahed, F., Cavuoto, L., & Chen, T. (2019). Predicting changes in driving safety performance on an individualized level under naturalistic driving conditions. 19th Annual Pilot Research Project Symposium, University of Cincinnati Education and Research Center, October 11-12, 2018, Cincinnati, Ohio, 1. | 2019 | 3 |
| 137 | Lerman, S. E., Eskin, E., Flower, D. J., George, E. C., Gerson, B., Hartenbaum, N., . . . Fati, A. P. T. F. (2012). Fatigue Risk Management in the Workplace. *Journal of Occupational and Environmental Medicine*, 54(2), 231-258. | 2012 | 1 |
| 138 | Li, W. (2016). A cabin crew fatigue risk comprehensive evaluation model. 12th International Conference on Fuzzy Systems and Knowledge Discovery (FSKD). | 2016 | 2 |
| 139 | Lillington, T., & McLeod, R. (2012). The challenge of fatigue management in a global company. Paper presented at the International Conference on Health, Safety and Environment in Oil and Gas Exploration and Production, Perth, Australia. | 2012 | 2 |
| 140 | Luckhaupt, S. E., Tak, S., & Calvert, G. M. J. S. (2010). The prevalence of short sleep duration by industry and occupation in the National Health Interview Survey. *Sleep.* 33(2), 149-159. | 2010 | 3 |
| 141 | Mabbott, N. (2003). ARRB pro-active fatigue management system. *Road and Transport Research*, 12(1), 56-62. | 2003 | 3 |
| 142 | Mani, A., Musolin, K., James, K., Kincer, G., Alexander, B., Succop, P., . . . Bhattacharya, A. (2014). Risk factors associated with live fire training: Buildup of heat stress and fatigue, recovery and role of micro-breaks. *Occupational Ergonomics*, 11(2-3), 109-121. | 2014 | 3 |
| 143 | Martell, M. (2018). Mine worker fatigue and circadian rhythms.: how biological clocks respond to light and darkness. *Coal Age*, 123(4), 38-39. | 2018 | 3 |
| 144 | McCauley, P., Kalachev, L. V., Mollicone, D. J., Banks, S., Dinges, D. F., & Van Dongen, H. P. (2013). Dynamic circadian modulation in a biomathematical model for the effects of sleep and sleep loss on waking neurobehavioral performance. *Sleep*, 36(12), 1987-1997 | 2013 | 3 |
| 145 | McDonald, J., Patel, D., & Belenky, G. (2010). Sleep and Performance Monitoring in the Workplace: The Basis for Fatigue Risk Management. In Principles and Practice of Sleep Medicine: Fifth Edition (pp. 775-783). | 2010 | 1 |
| 146 | Meade, D., & Tate, D. (2004). Specialized driver training: Elevating defensive driving from a simple awareness to a proactive, crash-free reality. SPE International Conference on Health, Safety, and Environment in Oil and Gas Exploration and Production, Calgary, Alberta, Canada. | 2004 | 3 |
| 147 | Mironov, A. D., & Nakvasin, A. Y. (2014). Development and flight research of non-intrusive airborne system of pilot psychophysiological status monitoring. 29^th^ Congress of the International Council of the Aeronautical Sciences, St Petersburg, Russia. | 2014 | 3 |
| 148 | Mirowski, L., & Scanlan, J. (2018) Mobile application based heavy vehicle fatigue compliance in Australian operations. In: Vol. 10837 LNCS (pp. 303-308). | 2018 | 3 |
| 149 | Mollicone, D., Kan, K., Mott, C., Bartels, R., Bruneau, S., van Wollen, M., . . . Van Dongen, H. P. A. (2019). Predicting performance and safety based on driver fatigue. *Accident Analysis and Prevention*, 126, 142-145. | 2019 | 3 |
| 150 | Moore-Ede, M., Heitmann, A., Guttkuhn, R., Trutschel, U., Aguirre, A., & Croke, D. (2004). Circadian Allertness Simulator for Fatigue Risk Assessment in Transportation: Application to Reduce Frequency and Severity of Truck Accidents. *Aviation Space and Environmental Medicine*, 75(3), A107-A118. | 2004 | 3 |
| 151 | Morris, M. B., Wiedbusch, M. D., & Gunzelmann, G. (2018). Fatigue Incident Antecedents, Consequences, and Aviation Operational Risk Management Resources. *Aerospace Medicine and Human Performance*, 89(8), 708-716. | 2018 | 3 |
| 152 | National Heavy Vehicle Regulator. (2016). Company A: Example Safety Case. | 2016 | 1 |
| 153 | Nix, S., & Brunette, S. (2015). Rest, Shift Duration, and Air Medical Crewmember Fatigue. *Air Medical Journal*, 34(5), 289-291. | 2015 | 3 |
| 154 | O'Connor, M., & Lincoln, J. (2013). Self-reported flight crew fatigue in commercial airline operations, 2009-2011. *Aviat Space Environ Med*, 84(4), 339. | 2013 | 4 |
| 155 | Olbert, Å. (2012). Flight and duty time limitations: A study on their effectiveness in managing crew fatigue. 52^nd^ AGIFORS Annual Proceedings, Phoenix, Arizona. | 2012 | 3 |
| 156 | Orasanu, J., Nesthus, T. E., Parke, B., Hobbs, A., Dulchinos, V., Kraft, N. O., . . . Mallis, M. (2011). Work schedules and fatigue management strategies in air traffic control (ATC). Proceedings of the Human Factors and Ergonomics Society Annual Meeting. | 2011 | 4 |
| 157 | Patterson, P. D., Moore, C. G., Weaver, M. D., Buysse, D. J., Suffoletto, B. P., Callaway, C. W., & Yealy, D. M. (2014). Mobile phone text messaging intervention to improve alertness and reduce sleepiness and fatigue during shiftwork among emergency medicine clinicians: study protocol for the SleepTrackTXT pilot randomized controlled trial. *Trials*, 15. | 2014 | 3 |
| 158 | Petrilli, R. M., Roach, G. D., Dawson, D., & Lamond, N. (2006). The sleep, subjective fatigue, and sustained attention of commercial airline pilots during an international pattern. *Chronobiology International*, 23(6), 1347-1362. | 2006 | 3 |
| 159 | Phillips, R. O., Kecklund, G., Anund, A., & Sallinen, M. (2017). Fatigue in transport: a review of exposure, risks, checks and controls. *Transport Reviews*, 37(6), 742-766. | 2017 | 2 |
| 160 | Pierre, B., Mathieu, J., Alexandre, G., & Anne-Sophie, N. (2019). Fatigue-related risk perception among emergency physicians working extended shifts. *Applied Ergonomics*, 82, 102914. | 2019 | 3 |
| 161 | Pruchnicki, S. A., Wu, L. J., & Belenky, G. (2011). An exploration of the utility of mathematical modeling predicting fatigue from sleep/wake history and circadian phase applied in accident analysis and prevention: The crash of Comair Fight 5191. *Accident Analysis and Prevention*, 43(3), 1056-1061. | 2011 | 3 |
| 162 | Queensland Department of Health. (2014). Human Resources Policy: Fatigue risk management. | 2014 | 2 |
| 163 | Queensland Government. (2012). Fatigue risk management system resource pack. | 2012 | 2 |
| 164 | Rajaratnam, S. M. W., Barger, L. K., Lockley, S. W., Shea, S. A., Wang, W., Landrigan, C. P., . . . Czeisler, C. A. (2012). Sleep disorders, health, and safety in police officers. *Journal of the American Medical Association*, 306(23), 2567-2578. | 2012 | 2 |
| 165 | Rangan, S., & Van Dongen, H. P. A. (2013). Quantifying Fatigue Risk in Model-Based Fatigue Risk Management. *Aviation Space and Environmental Medicine*, 84(2), 155-157. | 2013 | 3 |
| 166 | Redeker, N. S., Caruso, C. C., Hashmi, S. D., Mullington, J. M., Grandner, M., & Morgenthaler, T. I. (2019). Workplace interventions to promote sleep health and an alert, healthy workforce. *Journal of Clinical Sleep Medicine*, 15(4), 649-657. | 2019 | 1 |
| 167 | Reifman, J., Kumar, K., Wesensten, N. J., Tountas, N. A., Balkin, T. J., & Ramakrishnan, S. (2016). 2B-Alert Web: An Open-Access Tool for Predicting the Effects of Sleep/Wake Schedules and Caffeine Consumption on Neurobehavioral Performance. *Sleep*, 39(12), 2157-2159. | 2016 | 4 |
| 168 | Riethmeister, V., Brouwer, S., van der Klink, J., & Bultmann, U. (2016). Work, eat and sleep: towards a healthy ageing at work program offshore. *BMC Public Health*, 16. | 2016 | 3 |
| 169 | Riethmeister, V., Matthews, R. W., Dawson, D., de Boer, M. R., Brouwer, S., & Bültmann, U. (2019). Time-of-day and days-on-shift predict increased fatigue over two-week offshore day-shifts. *Applied Ergonomics*, 78, 157-163. | 2019 | 3 |
| 170 | Roach, G. D., Roberts, P., Dawson, D., Ferguson, S., Meuleners, L., Brook, L., & Sargent, C. (2017). Controlling fatigue risk in safety-critical workplaces: A summary of selected papers from the 9th International Conference on Managing Fatigue in Transportation, Resources and Health. *Accident Analysis and Prevention*, 99, 379-382. | 2017 | 1 |
| 171 | Roach, G. D., Sargent, C., Darwent, D., & Dawson, D. (2012). Duty periods with early start times restrict the amount of sleep obtained by short-haul airline pilots. *Accident Analysis and Prevention*, 45, 22-26. | 2012 | 3 |
| 172 | Romig, E., & Klemets, T. (2009). Fatigue Risk Management in Flight Crew Scheduling. *Aviation Space and Environmental Medicine*, 80(12), 1073-1074. | 2009 | 2 |
| 173 | Rosa, R. R. (2004). Commentary on a model to predict work-related fatigue based on hours of work. *Aviat Space Environ Med*, 75(3)(1), A72-A73. | 2004 | 1 |
| 174 | Rosa, R. R., & Colligan, M. J. (1992). Application of a portable test battery in the assessment of fatigue in laboratory and worksite studies of 12-hour shifts. *Scand J Work Environ Health*, 18(Suppl 2), 113-115. | 1992 | 3 |
| 175 | Ross, J. (2008). Digging deeper report and action plan. AusIMM Bulletin(5), 55. | 2008 | 1 |
| 176 | Rudari, L., Sperlak, L. A., Geske, R. C., Jones, G. E., III, & Johnson, M. E. (2014). The sustainability of FAR part 117: Flight and duty limitation and rest requirements for flight crewmembers. Proceedings of the Human Factors and Ergonomics Society Annual Meeting. | 2014 | 2 |
| 177 | Rudin-Brown, C. M., Harris, S., & Rosberg, A. (2019). How shift scheduling practices contribute to fatigue amongst freight rail operating employees: Findings from Canadian accident investigations. *Accident Analysis and Prevention*, 126, 64-69. | 2019 | 3 |
| 178 | Ryan, N. D., & Abassi, H. (2012). Fatigue risk management program - A fit for purpose approach. International Conference on Health, Safety and Environment in Oil and Gas Exploration and Production, Perth, Australia. | 2012 | 1 |
| 179 | Sagherian, K., Zhu, S., Storr, C., Hinds, P. S., Derickson, D., & Geiger-Brown, J. (2018). Bio-mathematical fatigue models predict sickness absence in hospital nurses: An 18 months retrospective cohort study. *Applied Ergonomics*, 73, 42-47. | 2018 | 3 |
| 180 | Sallinen, M., Sihvola, M., Puttonen, S., Ketola, K., Tuori, A., Harma, M., . . . Akerstedt, T. (2017). Sleep, alertness and alertness management among commercial airline pilots on short-haul and long-haul flights. *Accident Analysis and Prevention*, 98, 320-329. | 2017 | 4 |
| 181 | Santos, H. G., Chiavegato, L. D., Valentim, D. P., Da Silva, P. R., & Padula, R. S. (2016). Resistance training program for fatigue management in the workplace: exercise protocol in a cluster randomized controlled trial. *BMC Public Health*, 16(1), 1-11. | 2016 | 3 |
| 182 | Sargent, C., Darwent, D., Ferguson, S. A., & Roach, G. D. (2012). Can a simple balance task be used to assess fitness for duty? *Accident Analysis and Prevention*, 45, 74-79. | 2012 | 3 |
| 183 | Sather, T. E., & Delorey, D. R. (2016). Energy Beverage Consumption Among Naval Aviation Candidates. *Aerospace Medicine and Human Performance*, 87(6), 557-564. | 2016 | 3 |
| 184 | Schlesinger, D. (2017). FRA training requirements. ASME / IEEE Joint Rail Conference. | 2017 | 2 |
| 185 | Scott, L. D., Hofmeister, N., Rogness, N., & Rogers, A. E. (2010). Implementing a Fatigue Countermeasures Program for Nurses A Focus Group Analysis. *Journal of Nursing Administration*, 40(5), 233-240. | 2010 | 3 |
| 186 | Scott, L. D., Hofmeister, N., Rogness, N., & Rogers, A. E. (2010). An Interventional Approach for Patient and Nurse Safety A Fatigue Countermeasures Feasibility Study. *Nursing Research*, 59(4), 250-258. | 2010 | 3 |
| 187 | Sharwood, L. N., Elkington, J., Meuleners, L., Ivers, R., Boufous, S., & Stevenson, M. (2013). Use of caffeinated substances and risk of crashes in long distance drivers of commercial vehicles: case-control study. *British Medical Journal*, 346. | 2013 | 3 |
| 188 | Shaw, A., Blewett, V., & Aickin, C. (2007). Digging deeper - OHS in the NSW mining industry. | 2007 | 2 |
| 189 | Signal, T. L., Ratieta, D., & Gander, P. H. (2008). Flight crew fatigue management in a more flexible regulatory environment: An overview of the New Zealand aviation industry. *Chronobiology International*, 25(2-3), 373-388. | 2008 | 2 |
| 190 | Smiley, A. (2015). Chapter 11: Fatigue and Driving. In A. Smiley (Ed.), Human Factors and Traffic Safety (3 ed.). Tucson, Arizona: Lawyers & Judges Publishing Company, Inc. | 2015 | 2 |
| 191 | Smiley, A., Smahel, T., Boivin, D., Boudreau, P., Remmers, J., Turner, M., . . . Gregory, K. (2010). Summary report Phase III: Effects of a fatigue management program on fatigue in the commercial motor carrier industry Retrieved from Montreal, QC, Canada: | 2010 | 5 |
| 192 | Smiley, A. M. (1990). The Hinton train disaster. *Accident Analysis & Prevention*, 22(5), 443-455. | 1990 | 2 |
| 193 | Smith, M. R., & Eastman, C. I. (2012). Shift work: Health, performance and safety problems, traditional countermeasures, and innovative management strategies to reduce circadian misalignment. *Nature and Science of Sleep*, 4, 111-132. | 2012 | 1 |
| 194 | Sparrow, A. R., Mollicone, D. J., Kan, K., Bartels, R., Satterfield, B. C., Riedy, S. M., . . . Van Dongen, H. P. A. (2016). Naturalistic field study of the restart break in US commercial motor vehicle drivers: Truck driving, sleep, and fatigue. *Accident Analysis and Prevention*, 93, 55-64. | 2016 | 3 |
| 195 | Sparrow, A. R., Riedy, S., & Van Dongen, H. P. (2017). Fatigue risk management by prior sleep wake model (PSWM): Too easy to be reliable? Sleep, 40, A57-A57. | 2017 | 3 |
| 196 | Steege, L. M., Pasupathy, K. S., & Drake, D. (2014). Relationships between wellness, fatigue, and intershift recovery in hospital nurses. Proceedings of the Human Factors and Ergonomics Society Annual Meeting 58(1):778-782 | 2014 | 3 |
| 197 | Steege, L. M., & Pinekenstein, B. (2016). Addressing occupational fatigue in nurses a risk management model for nurse executives. Journal of Nursing Administration, 46(4), 193-200. | 2016 | 3 |
| 198 | Steege, L. M., Pinekenstein, B. J., Rainbow, J. G., & Knudsen, E. A. (2017). Addressing Occupational Fatigue in Nurses Current State of Fatigue Risk Management in Hospitals, Part 2. *Journal of Nursing Administration*, 47(10), 484-490. | 2017 | 3 |
| 199 | Stewart, S., Holmes, A., Jackson, P., & Abboud, R. (2006). An integrated system for managing fatigue risk within a low cost carrier. 59^th^ Annual International Air Safety Seminar (IASS), Paris, France. | 2006 | 1 |
| 200 | Suh, Y. A., Kim, J. H., & Yim, M. S. (2019). An Investigation into the Feasibility of Monitoring a Worker's Psychological Distress. In T. Z. Ahram (Ed.), Advances in Artificial Intelligence, Software and Systems Engineering (Vol. 787, pp. 476-487). | 2019 | 3 |
| 201 | Sun, R. S., Song, W. S., Li, J. Q., & Tian, W. L. (2014). Study on a Model of Flight Fatigue Dynamic Risk Index. In D. Harris (Ed.), Engineering Psychology and Cognitive Ergonomics, Epce 2014 (Vol. 8532, pp. 375-386). | 2014 | 3 |
| 202 | Taylor, T. S., Watling, C. J., Teunissen, P. W., Dornan, T., & Lingard, L. (2016). Principles of fatigue in residency education: a qualitative study. CMAJ Open, 4(2), E200-204. | 2016 | 3 |
| 203 | Theron, W. J., & van Heerden, G. M. J. (2011). Fatigue knowledge-a new lever in safety management. *Journal of the South African Institute of Mining and Metallurgy*, 111(1), 1-10. | 2011 | 3 |
| 204 | Thomas, M. J. W., & Ferguson, S. A. (2010). Prior Sleep, Prior Wake, and Crew Performance During Normal Flight Operations. Aviation Space and Environmental Medicine, 81(7), 665-670. | 2010 | 3 |
| 205 | Thomas, M. J. W., Paterson, J. L., Jay, S. M., Matthews, R. W., & Ferguson, S. A. (2019). More than hours of work: fatigue management during high-intensity maritime operations. Chronobiology International, 36(1), 143-149. | 2019 | 3 |
| 206 | Transportation Safety Board of Canada. (2015). Rail Transportation Safety Investigation R13T0192. | 2015 | 2 |
| 207 | Trutschel, U., Guttkuhn, R., Heitmann, A., Aguirre, A., & Moore-Ede, M. (2003). Expert System for simulating and predicting sleep and alertness patterns. In V. Palade, R. J. Howlett, & L. Jain (Eds.), Knowledge-Based Intelligent Information and Engineering Systems, Pt 1, Proceedings (Vol. 2773, pp. 104-110). | 2003 | 3 |
| 208 | Tsai, M. K. (2017). Enhancing nuclear power plant safety via on-site mental fatigue management. *Nuclear Technology and Radiation Protection*, 32(1), 109-114. | 2017 | 3 |
| 209 | Tucker, P., Lombardi, D., Smith, L., & Folkard, S. (2007). The impact of rest breaks on temporal trends in injury risk. *Chronobiol Int*, 23(6), 1423-1434. | 2007 | 3 |
| 210 | van den Berg, M. J., Signal, T. L., Mulrine, H. M., Smith, A. A. T., Gander, P. H., & Serfontein, W. (2015). Monitoring and Managing Cabin Crew Sleep and Fatigue During an Ultra-Long Range Trip. *Aerospace Medicine and Human Performance*, 86(8), 705-713. | 2015 | 3 |
| 211 | van den Berg, M. J., Wu, L. J., & Gander, P. H. (2016). Subjective Measurements of In-Flight Sleep, Circadian Variation, and Their Relationship with Fatigue. *Aerospace Medicine and Human Performance*, 87(10), 869-875. | 2016 | 3 |
| 212 | Van Dongen, H. P. A. (2010). Predicting Sleep/Wake Behavior for Model-Based Fatigue Risk Management. *Sleep*, 33(2), 144-145. | 2010 | 3 |
| 213 | Van Dongen, H. P. A. (2018). Evidence-Based Guidelines for Fatigue Risk Management in Emergency Medical Services: A Significant Step Forward and a Model for Other High-Risk Industries. *Prehospital Emergency Care*, 22, 110-112. | 2018 | 2 |
| 214 | Van Dongen, H. P. A., Belenky, G., & Vila, B. J. (2011). The Efficacy of a Restart Break for Recycling with Optimal Performance Depends Critically on Circadian Timing. *Sleep*, 34(7), 917-929. | 2011 | 3 |
| 215 | Van Dongen, H. P. A., Mott, C. G., Huang, J. K., Mollicone, D. J., McKenzie, F. D., & Dinges, D. F. (2007). Optimization of biomathematical model predictions for cognitive performance impairment in individuals: Accounting for unknown traits and uncertain states in homeostatic and circadian processes. *Sleep*, 30(9), 1129-1143. | 2007 | 3 |
| 216 | van Drongelen, A., van der Beek, A. J., Hlobil, H., Smid, T., & Boot, C. R. L. (2013). Development and evaluation of an intervention aiming to reduce fatigue in airline pilots: design of a randomised controlled trial. BMC Public Health, 13. | 2013 | 3 |
| 217 | Vejvoda, M., Elmenhorst, E. M., Pennig, S., Plath, G., Maass, H., Tritschler, K., . . . Aeschbach, D. (2014). Significance of time awake for predicting pilots' fatigue on short-haul flights: implications for flight duty time regulations. *Journal of Sleep Research*, 23(5), 564-567. | 2014 | 3 |
| 218 | Vila, B., Morrison, G. B., & Kenney, D. J. (2002). Improving Shift Schedule and Work-Hour Policies and Practices to Increase Police Officer Performance, Health, and Safety. *Police Quarterly*, 5(1), 4-24. | 2002 | 3 |
| 219 | Vincent, G. E., Aisbett, B., Wolkow, A., Jay, S. M., Ridgers, N. D., & Ferguson, S. A. (2018). Sleep in wildland firefighters: what do we know and why does it matter? *International Journal of Wildland Fire*, 27(2), 73-84. | 2018 | 2 |
| 220 | Vo, S. A., Mirowski, L., Scanlan, J., & Turner, P. (2019). Verifying driver performance for heavy haulage fatigue management. *IET Intelligent Transport Systems*, 13(6), 1033-1040. | 2019 | 3 |
| 221 | Vo, S. A., Scanlan, J., Mirowski, L., & Turner, P. (2019) An approach to verify heavy vehicle driver fatigue compliance under Australian chain of responsibility regulations. In: Vol. 886 (pp. 584-599). | 2019 | 2 |
| 222 | Waclawski, E., & Noone, P. (2017). Are aviation industry fatigue risk management strategies needed in healthcare? *Anaesthesia*, 72(11), 1417-1419. | 2017 | 2 |
| 223 | Wang, L., & Sun, R. (2013). A remote and real-time fatigue measurement solution based on eye tracking technique. *Information Technology Journal*, 12(21), 6050-6055. | 2013 | 3 |
| 224 | Wang, Z., Zhang, H., Zhang, Q., & Li, S. (2018). Interactive Effect of Circadian Rhythm and Time on Task on Driver Fatigue Level. 18th COTA International Conference of Transportation. | 2018 | 3 |
| 225 | Williamson, A. (2007). Predictors of psychostimulant use by long-distance truck drivers. *American Journal of Epidemiology*, 166(11), 1320-1326. | 2007 | 3 |
| 226 | Williamson, A., Friswell, R., Grzebieta, R., & Olivier, J. (2013). What do we tell drivers about fatigue management? International Conference on Contemporary Ergonomics and Human Factors 2013; Cambridge; United Kingdom. | 2013 | 2 |
| 227 | Wong, I. S., Popkin, S., & Folkard, S. (2019). Working Time Society consensus statements: A multi-level approach to managing occupational sleep-related fatigue. *Industrial Health*, 57(2), 228-244. | 2019 | 1 |
| 228 | Wong, L. R., Flynn-Evans, E., & Ruskin, K. J. (2018). Fatigue Risk Management: The Impact of Anesthesiology Residents' Work Schedules on Job Performance and a Review of Potential Countermeasures. *Anesthesia and Analgesia*, 126(4), 1340-1348. | 2018 | 3 |
| 229 | Wu, L. J. (2014). Evidence based fatigue risk management during 24/7 operations: Objective assessment of pilots' sleep, performance, and fatigue during ultra long range and long range flights (Doctoral dissertation). | 2014 | 3 |
| 230 | Wu, L. J., Gander, P. H., van den Berg, M., & Signal, T. L. (2018). Equivalence Testing as a Tool for Fatigue Risk Management in Aviation. *Aerospace Medicine and Human Performance*, 89(4), 383-388. | 2018 | 3 |
| 231 | Yaakob, N. M., & Abdullah, L. A. (2019). Leveraging technology to manage health, safety and human rights risks: A differentiated approach in managing fatigue. SPE Symposium: Asia Pacific Health, Safety, Security, Environment and Social Responsibility, Kuala Lumpur, Malaysia. | 2019 | 3 |

1. Supplementary Material References

1. IATA, ICAO, and IFAPLA., *Fatigue Risk Management Systems - Implementation Guide for Operators*. 2011, Montreal, Canada: IATA.

2. International Air Transport Association, *Fatigue Safety Performance Indicators (SPIs): A Key Component of Proactive Fatigue Hazard Identification*. 2014, Montreal, Canada: IATA.

3. Higgins, J.P., et al., *The Cochrane Collaboration’s tool for assessing risk of bias in randomised trials.* Bmj, 2011. **343**.
